# In the thick of it: scope rivalry in past counterfactuals of Pomerano

**DOI:** 10.1007/s10828-022-09137-9

**Published:** 2022-12-12

**Authors:** Göz Kaufmann

**Affiliations:** German Department, Albert-Ludwigs-Universität Freiburg, Freiburg im Breisgau, Germany

**Keywords:** Pomerano, Past counterfactuals with modal verbs, Scope rivalry, Verb clusters in CP, Blocking of tense and/or person agreement, Weak/strong probes

## Abstract

This paper analyzes the morphosyntactic variation in past counterfactuals with modal verbs in Pomerano, a Low German variety spoken in Brazil. The variation concerns (i) the highest verb (temporal auxiliary or modal verb), (ii) the morphological form of the temporal auxiliary (blocking of tense and/or person agreement), (iii) the frequently unexpected position of the modal verb (verb clusters in the CP-domain), and (iv) the overall number of verbs (syntactic doubling and/or PF-insertion). Analyzing more than 6,000 translated sentences, scope rivalry between the temporal auxiliary and the modal verb proves to be the major catalyst of an intriguing instance of language variation and change. The derivation of the extant variants grants us a privileged view of the clausal architecture of Pomerano—including cases of derivational misfiring—as well as of more general processes of clause formation.

## Introduction

This paper analyzes the manifold ways in which speakers of Pomerano, a Low German variety spoken in several parts of Brazil, code past counterfactuals with modal verbs (PCF+MVs). By comparing this clause type with past counterfactuals without modal verbs (PCF-MVs), a clause type that does not display the same amount of variation, we follow Iatridou’s (2000, 231) core inquiry into “how the form of counterfactuals is related to their meaning.” Following Salzmann (2019) and Embick and Noyer (2007), we will derive the form of PCF+MVs in syntax proper as long as there is a direct relationship to semantics. With regard to morphology, we will work within the framework of Distributed Morphology (DM), thus favoring a post-syntactic approach. Importantly, unlike many research projects in the generative framework, we will base our conclusions on quantitative analyses of a robust data set of more than 6,000 sentences that were translated by 104 informants. This said, we present a PCF-MV in (1):

**Table:** 

stimulus <64>	Portuguese: Se ele tivesse assinado esse contrato, ele teria perdido muito dinheiro.						
	English: If he had signed this contract, he would have lost a lot of money.						
(1)	Wen	hai	dai	kontrat	unnersreewa	ha,	den
	*if*	*he.3SG.NOM*	*the*	*contract*	*signed.PP*	*had.3SG.PST*	then

	ha	hai	seir	feel	gild:	forloora.^1^	
	*had.3SG.PST*	*he.3SG.NOM*	very	*much*	*money*	*lost.PP*	
					(Pom-154; f/38/Pom)^2^		

The speaker of (1) speculates about what could have happened (*a male person losing a lot of money*) if the thing that did not happen (*this person signing a specific contract*) had happened. In view of the cognitive complexity of this PCF-MV,^3^ its morphosyntax may, at first, appear somewhat undercomplex. One must not forget though that Pomerano does not differ substantially from English in this respect and that it possesses all features Iatridou (2000) describes for past counterfactuals. Just like English, Pomerano uses a pluperfect in the antecedent, which according to Iatridou (2000, 240) “contain[s] two levels of past.” It “uses one of its ‘past’ layers for CF [counterfactual] purposes and the other for temporal purposes.” Iatridou (2000) only relates the counterfactual layer directly to the ‘past tense’ morphology of the temporal auxiliary (TA). In the English stimulus version of (1), presented here as (2a), *had* fulfills this function. By comparing (2a) to its present counterfactual equivalence in (2b), we can identify the temporal layer.

**Table:** 

(2)	a.	If he had.3SG.PST signed.PP this contract, he would.3SG.PST have.INF lost.PP a lot of money.
	b.	If he signed.3SG.PST this contract, he would.3SG.PST lose.INF a lot of money.

Both sentences feature ‘past tense’ morphology. As (2b) does not code a past event, Iatridou (2000) calls the ‘past tense’ morphology in *signed* an “exclusion feature”. Counterfactuals such as (2a–b) apply this feature in *had*, *signed*, and *would*, etymologically the past tense form of *will*, to describe a topic world that excludes the actual world. It thus ranges over worlds, not over times as in the factual clause *He signed the contract*.

The question now arises of how the temporal layer is expressed in (2a), which does not only exclude the actual world, but also the utterance time. In Iatridou’s (2000, 246) terminology, (2a) “expresses a temporal relation of precedence between the topic time and the utterance time.” Comparing the morphosyntax of (2a–b), it stands to reason that the past participles *signed* and *lost* in (2a) possess a past/perfect feature and express precedence (cf. Grewendorf 1995, but also McFadden and Alexiadou 2005,4). This conclusion can be straightforwardly extended to (1).

Importantly, according to Iatridou (2000), the past subjunctive mood in Standard German (StG) or Italian past counterfactuals is not decisive. She (2000, 266 and 266, fn. 39) considers the subjunctive a “condition on the PF branch”, adding that “[t]he semantics of some other element brings about the particular meaning, but the subjunctive is a well-formedness condition.” Unlike StG, Pomerano does not possess a productive subjunctive mood.

The consequent of (1) also uses the pluperfect. In this, Pomerano differs from English, which uses *would* plus a perfect infinitive, and thus combines a future marker (*will*) with the exclusion feature expressed by ‘past tense’ morphology (cf. Iatridou 2000, 233). However, one may consider the resumptive adverb *den* ‘then’ in the consequent of (1), which appears in roughly 53% of the conditional compound sentences in the Pomerano data set, as a non-obligatory marker for its future/subsequent quality.^5^ The unspectacular morphosyntax of (1) changes dramatically when the stimulus sentence features an additional form of counterfactuality, a modal verb (MV; cf. Kulakova and Nieuwland 2016, 61):

**Table:** 

stimulus <20>	Portuguese: Se ele tivesse podido consertar o carro, ele teria feito isso.						
	English: If he could have repaired the car, he would have done it.						
(3)	Wen	hai	hät	küüt	dai	auto	heilmåkt
	*if*	*he.3SG.NOM*	*has.3SG.PRS*	*can.PP*	*the*	*car*	*repaired.PP*
	häwa,	den	had	hai	dat	ook	måkt.
	*have.INF*	then	*had.3SG.PST*	*he.3SG.NOM*	*it*	PRT	*made.PP*
						(Pom-221; m/18/Pom+Port)	

The consequent of (3) does not contain a MV and displays the same morphosyntax as (1), the TA *had* ‘had’ and the past participle *måkt*^6^ ‘made’. Granted, finite *had*^7^ in (3) differs from *ha* in (1), but this is merely a case of phonetic variation, as ‘tense’ in the TA is expressed by the vowel, as can be seen in Postma’s (2019, 107) paradigm for *häwa* in Pomerano from Espírito Santo (ES) (cf. Table 1).

**Table 1:** Paradigm of *häwa* ‘have’ according to Postma (2019, 107; with correction of one typo)

Present tense		Past tense		Past participle
1SG	ik hä(w)	1SG	ik haar	hat
2SG	duu häst	2SG	duu haarst	
3SG	hai hät	3SG	hai haar	
1–3PL	wij/jij/sai häwa	1–3PL	wij/jij/sai haara	

The first column shows the present tense singular and plural forms. All these forms feature the vowel [ɛ] written as <ä>. The second column lists the corresponding ‘past tense’ forms, all with the vowel [a:] written as <aa>. The last column illustrates the past participle *hat*. Although Postma (2019) offers an extremely insightful description of the grammar of Pomerano in ES, it should not surprise the reader that such a grammatical overview cannot do justice to the actual variation in a language without an established standard variety. This paper will demonstrate that Pomerano—at least in RS—is more complex than Table 1 suggests. On the one hand, there are several phonetic variants for the same position of the paradigm; on the other hand, the appearance of (a) certain variant(s) depend(s) heavily on the speaker group and the clausal context. Thus, Table 1 may represent factual contexts well, but falls short of PCF+MVs.

The crucial difference between (1) and (3) concerns the antecedent. Translation (3) features a MV and contains two conspicuous morphosyntactic characteristics not present in (1). (i) Although the TA *hät* ‘has’ may be said to agree with the 3SG-subject pronoun *hai* ‘he’, it unexpectedly appears with present tense morphology. (ii) The antecedent in (3) features two appearances of the TA, first as finite *hät* and then as infinitive *häwa*. Each of these forms selects a past participle. The MV *küüt* ‘can.PP’ is governed by *hät*, the main verb *heilmåkt* ‘repaired.PP’ by *häwa*. With this, the first two questions this paper intends to answer can be formulated:

(i) Why does the TA *hät* appear with present tense morphology in the antecedent of (3), i.e., what blocks the expected ‘past tense’ morphology present in (1) and in the consequent of (3)?
(ii) Does the blocking of ‘past tense’ morphology in the TA cause the appearance of a second past participle, i.e., does the additional participle *heilmåkt* ‘repaired’ express one of the two levels of past of PCF+MVs?

In our view, the blocking of ‘tense’ on the TA mentioned in (i) is caused by the presence of the MV. If question (ii) is answered in the positive, past participles in Pomerano contain an exclusion feature just like the ‘past tense’ forms *ha*/*had* in (1) and (3). The higher past participle in the antecedent of (3) would then exclude the actual world, while the lower one would exclude the utterance time. Counterfactual idiosyncrasies of Pomerano do not end with (3). Some of the 78 relevant translations of sentence <20> feature the MV *koina* ‘can’ in both the antecedent and the consequent. This deviation causes further morphosyntactic changes as illustrated in (4a–b):

**Table:** 

stimulus <20>		Portuguese: Se ele tivesse podido consertar o carro, ele teria feito isso.							
		English: If he could have repaired the car, he would have done it.							
(4)	a.	Wen	hai	häär	küüt	dai	auto	trechtmåkt	häwa,
		*if*	*he.3SG.NOM*	*has.3SG.PRS*	*can.PP*	*the*	*car*	*repaired.PP*	*have.INF*
		(0.3)	den	häär	küüt	hai	dat	måkt	häwa.
		*(0.3)*	then	*has.3SG.PRS*	can*.PP*	*he.3SG.NOM*	*it*	*made.PP*	*have.INF*
								(Pom-143; f/16/Pom)	
	b.	Wen	hai	hä	küüt	mine	auto	måkt	häwa,
		*if*	*he.3SG.NOM*	*has.3SG.PRS*	*can.PP*	*my*	*car*	*made.PP*	*have.INF*
		den	küüt	hai	dat	ook	måkt	hat	häwa.
		then	*can.PP*	*he.3SG.NOM*	*it*	PRT	*made.PP*	*had.PP*	*have.INF*
								(Pom-108; m/54/Pom)	

The finite TAs *häär* and *hä* in the antecedents of (4a–b) are mere phonetic variants of *hät* in (3). It is their consequents that offer new fascinating characteristics. In (4a), the initial sequence *den* ***häär küüt*** *hai* constitutes a syntactic *rarum* in Continental West Germanic varieties. If the unstressed subject pronoun *hai* marks the topological border between the left clausal bracket, the head of CP, and the midfield, the IP-domain, its position suggests the presence of two verbs in the CP-domain. Postma (2014, 639–642), who described this phenomenon for P(C)F+MVs from ES, calls this “V2 of verbal clusters”. The decisive role of the MV in this construction can be deduced from the fact that “V2 of verbal clusters” is only instantiated in the combination of a TA and a MV (cf. Postma 2014, 639, fn. 19).

In (4b), the past participle *küüt* is the only verb in the CP-domain of the consequent. As a result, this clause does not possess any sign of finiteness. Moreover, a curious clause-final verbal triple *måkt hat häwa* ‘made had have’ with two additional past participles surfaces.^8^ Aside from this, the TA *hä* in the antecedent of (4b) is hardly audible. This happens in a few tokens and may represent a preliminary stage for the outright disappearance of the TA in the consequent of (4b). With (4a–b), four more questions arise. Two of them are concerned with the syntactic *rarum* of two verbs in the CP-domain; two of them deal with the phonetic shape of the TA:

(iii) Does Pomerano really allow two verbs in the CP-domain and what does this mean for the positions of preceding constituents such as *den* ‘then’ and subsequent constituents such as *hai* ‘he’ in (4a)?
(iv) If (iii) is answered in the positive, an ensuing question is what the derivational/semantic cause for the two verbs in the CP-domain is.
(v) Despite the fact that *hä*/*häär*/*hät* only represent phonetic variants of the present tense, the question arises of whether a phonetic erosion process *hät* > *häär* > *hä* > *ä* > *ø* exists. Such a process could help explain the non-finite consequent of (4b).
(vi) How does Pomerano cope with the lack of finiteness in a finite clause such as the consequent of (4b)?

These six questions will be dealt with in the rest of this paper. Section 2 offers basic facts about Pomerano and the Pomerano data set. Section 3 then sorts the Pomeranian informants into five groups of speakers according to their translation of one particular stimulus sentence. Section 4 interprets the different coding possibilities of PCF+MVs as subsequent stages in an instance of language change and describes their morphosyntax in detail. Section 5 presents the structural correlates for these coding possibilities, detailing the derivation of both the IP- and the CP-domain. Section 6 offers some conclusions and some further research possibilities.

## Pomerano in Brazil

### The language

In the aftermath of World War II, almost all speakers of Eastern Pomeranian varieties were expelled from the area east of the river Oder. Their dispersion throughout the rest of Germany and the ensuing language assimilation caused the wholesale disappearance of these varieties in Europe. However, many Eastern Pomeranians had left this continent in the 19th century. While smaller contingents migrated to Brazil, most of them headed for the United States. In spite of this, there are hardly any speakers left there. The rapid language shift to English resulted from a great deal of similarity to and contact with the English-speaking majority population. Migration to Brazil started in 1858 and lasted for 30 years. Unlike in the United States, the Lutherism of the Pomeranians in Brazil clashed with the dominant Catholic belief system. Furthermore, the language they spoke exhibited a more marked linguistic distance to the majority language Portuguese. The number of speakers of Pomerano, as Eastern Pomeranian varieties are called in Brazil, ranges from 200,000 to 250,000. Most of them live in the federal states of Rio Grande do Sul (RS), Espírito Santo (ES), Santa Catarina (SC), and Rondônia (RO). Importantly, hardly any speakers are fluent in StG, a fact that is directly related to the restrictive language laws of the *Estado Novo* (1937–1945), which prohibited, among other things, its use in the parochial school system. Although the loss of the StG roof might be considered one of the reasons that currently endanger the maintenance of Pomerano, its absence seems to have allowed for many intriguing changes, one of which is the topic of this paper (cf. Kaufmann and Duran in print for curious cases of phonetic variation).

### The Pomerano data set

The present study follows the elicitation method of the Mennonite Low German (MLG) data set, which is available from the IDS-archive for spoken German (cf. Kaufmann 2018). This data set is based on the translations of 46 English, Spanish, and Portuguese stimulus sentences into MLG by 321 Mennonite informants from North and South America. The stimulus sentences were read to the informants one by one, and the informants translated these sentences immediately and without the help of a written version. The project’s major goal was to study the serialization of clause-final verb clusters in different clause types. In order to guarantee clause-final clusters with two verbs in root clauses and clusters with three verbs in non-root clauses, nine PCF+MVs stimulus sentences and one stimulus sentence with an epistemic MV governing a perfect infinitive (cf. stimulus sentence <9> in (7)) were included.

As the MLG data set cast much light on diverse syntactic, morphological, and lexical phenomena (cf., e.g., Kaufmann 2007, 2015, 2017), twenty Pomeranians from RS were asked in 2013 to translate the 46 stimulus sentences. Since their translations contained many intriguing phenomena in PCF+MVs, 15 new sentences were added, among them several PCF+MVs and PCF-MVs. So far, the resulting 61 stimulus sentences have been translated by 104 informants from RS, 69 informants from ES, and 77 informants from RO. There are thus roughly 15,000 tokens available for analysis though the present paper focusses, with some exceptions, on the data from RS.

Both the Pomerano and the MLG data set offer two crucial advantages. First, the elicited data are comparable since all informants translated the same sentences. Second, the data allow detailed quantitative analyses of seemingly unrelated (syntactic) phenomena, for example, the concurrent presence of two adjacent verbs in the CP-domain and of a clause-final verbal triple, as in (5c) and (6) (cf. Table 8).

Aside from the translations, sociolinguistically relevant information was elicited,^9^ most importantly the informants’ competences in Pomerano, Portuguese, and StG (cf. fn. 2). Forty-four informants from RS claim to be more competent in Pomerano (42.3%), while 35 refer to Portuguese as their dominant language (33.7%). The remaining 25 informants consider themselves ambilingual (24%). Importantly, on average, even the 35 Portuguese-dominant speakers reach a solid 9.1 out of 14 possible points for their competence in Pomerano.

## Different coding strategies in PCF+MVs

Our initial interest in counterfactuality in Pomerano was aroused by the translations of stimulus sentence <45> *Ontem eu poderia ter vendido o anel* ‘Yesterday I could have sold the ring’. Despite its comparable cognitive complexity, sentence <45> is structurally less complex than sentence <20> (cf. (3) and (4a–b)). Due to this, it was translated with great ease by 101 of the 104 informants from RS and thus constitutes a good base for sorting the informants into five groups. Table 2 presents these groups, summarizing the core characteristics of their translations of sentence <45>.

**Table 2:**
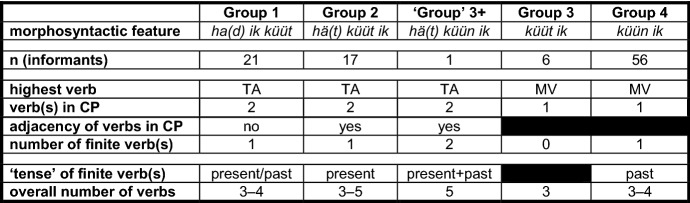
Core characteristics of five translation variants of stimulus sentence <45>

**Fig. 1:**
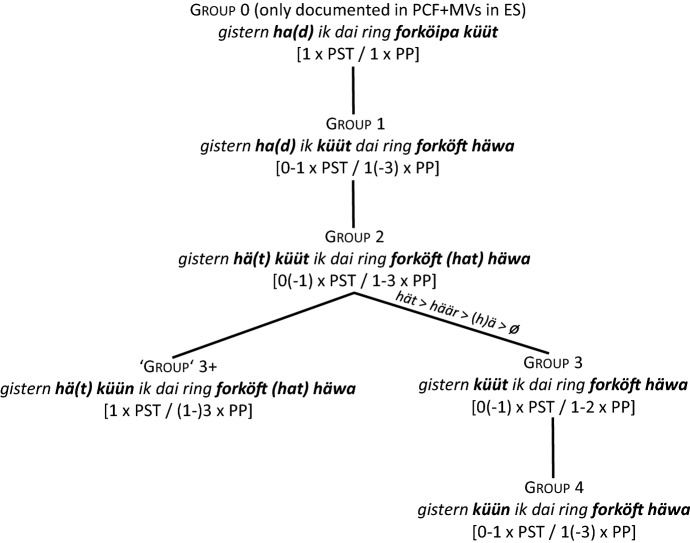
Subsequent stages in the coding of PCF+MVs in Pomerano

Each group is presented with its crucial morphosyntactic feature in the CP-domain of sentence <45> (*ha(d) ik küüt*, etc.). The groups are numbered according to the assumed stages of the morphosyntactic change of PCF+MVs in Pomerano (cf. Fig. 1). As one of the translation variants is only represented by a single speaker, its designation was put in quotation marks (cf. ‘Group’ 3+). The first four characteristics in Table 2 are decisive for grouping, the last two (‘tense’ of finite verb(s); overall number of verbs) are not. The first coding strategy we want to present features two adjacent verbs in the CP-domain (cf. the consequent of (4a)). It is represented by 17 group 2-speakers.

**Table:** 

stimulus <45>		Portuguese: Ontem eu poderia ter vendido o anel.						
		English: Yesterday I could have sold the ring.						
(5)	a.	Gistern	häär	küüt	ik	mijne	[0.6]^10^	fingerring
		*yesterday*	*has.3SG.PRS*	*can.PP*	*I.1SG.NOM*	*my*	*[0.6]*	*ring*
		forköipa.						
		*sell.INF*						
							(Pom-74; f/68/Pom)	
	b.	Gistern	hä	küüt	ik	mijne	fingerring	[1.0]
		*yesterday*	*has.3SG.PRS*	*can.PP*	*I.1SG.NOM*	*my*	*ring*	*[1.0]*
		forköft	häwa.					
		*sold.PP*	*have.INF*					
						(Pom-53; f/54/Port>Pom-86%)		
	c.	Gistern	hät	küüt	ik	dai	fingerring	forköft
		*yesterday*	*has.3SG.PRS*	*can.PP*	*I.1SG.NOM*	*the*	*ring*	*sold.PP*
		hat	häwa.					
		*had.PP*	*have.INF*					
						(Pom-206; f/51/Pom+Port)		

The CP-domain coincides for all group 2-speakers, but they display some variation with regard to the total number of verbs. In addition to the unique translation in (5a) with just one past participle, 12 informants produce two past participles, as in (5b), and four informants produce three past participles, as in (5c). The assumption that an additional past participle provides the necessary exclusion feature in a PCF+MV without ‘past tense’ morphology is supported by the fact that none of the 17 translations of group 2-speakers feature a TA with ‘past tense’ morphology, while 16 feature (an) additional past participle(s), as in (5b–c).

Aside from the blocking of ‘tense’ agreement, (5a–c) make it clear that person agreement is also blocked. The expected form of the TA would be *häw* (have.1SG.PRS), normally realized as *häf* (cf. Table 1). *Häf* occurs almost exclusively in translations with a 1SG-subject pronoun when it is a full verb or a TA in the present perfect tense. It does, however, not occur a single time in the group 2-speakers’ translations of sentence <45>. Further support for the assumption that it is indeed the MV that blocks ‘tense’ and person agreement of the TA comes from (6), which is only represented by a single ‘group’ 3+-speaker.

**Table:** 

stimulus <45>	Portuguese: Ontem eu poderia ter vendido o anel.							
	English: Yesterday I could have sold the ring.							
(6)	Gistern	hät	küün	ik	[0.3]	dai	anel	forköft
	*yesterday*	*has.3SG.PRS*	*can.1SG.PST*	*I.1SG.NOM*	*[0.3]*	*the*	*ring*	*sold.PP*
	hat	häwa.						
	*had.PP*	*have.INF*						
						(Pom-65; m/19/Pom+Port)		

Many of the characteristics described so far are shared by (6). The present tense form *hät*, the clause-final triple *forköft hat häwa* ‘sold had have’, and the unexpected double of TA and MV in the CP-domain. What supports the special role of the MV in general and especially in (6) is the fact that this verb, which is governed by *hät* and therefore expected to appear as a past participle, actually appears as the ‘past tense’ form *küün*. At first sight, *küün* could be regarded as a past participle by assuming—as in West Frisian—the existence of a weak participle *küüt* and a strong participle *küün*. However, Postma’s (2019, 109) paradigm for *koina* ‘can’ in Table 3 does not suggest such a solution.

**Table 3:** Paradigm of *koina* ‘can’ according to Postma (2019, 109; with corrections of some typos)

Present tense		Past tense		Past participle
1SG	ik ka	1SG	ik küü(n)	küüt
2SG	duu kast	2SG	duu küüst	
3SG	hai ka	3SG	hai küü(n)	
1–3PL	wij/jij/sai koina	1–3PL	wij/jij/sai küüna	

*Küün* only appears as a ‘past tense’ form, not as a past participle (cf. last column). As the 2SG-‘past tense’ form *küüst* is less ambiguous than *küün*, (7) should dispel any pending doubts with regard to the general possibility of double finiteness in Pomerano:

**Table:** 

stimulus<9>	Portuguese: Elisabete insiste que tu deves ter visto o caminhão.								
	English: Elisabeth insists that you must have seen the truck.								
(7)	Elisabete	sägt,		duu		häst	dai-	[0.4]	ha
	*Elisabeth*	*says*	*ø*	*you.2SG.NOM*		have*.2SG.PRS*	the-	*[0.4]*	*had.3SG.PST*
	küüst		dai	wåga	saia	häwa.			
	*can.2SG.PST*		*the*	*car*	*seen.PP*	*have.INF*			
							(Pom-517; f/48/Pom)		

Token (7) comes from a female speaker from RO and will not enter the analyses of the present paper. Its production process is nevertheless telling in that it demonstrates agreement blocking in action. The reader may object that stimulus sentence <9> is not a PCF+MV since it contains an epistemic MV. However, Pomerano does not distinguish morphosyntactically between these two types of modality. Informant Pom-517 first produces a TA *häst* that agrees with the 2SG-subject pronoun *duu* ‘you.2SG’ and displays present tense morphology. She probably does this in order to construct the present perfect tense, a frequent translation variant for sentence <9> (cf. the following definite article *dai* probably initializing the DP *dai wåga* ‘the car’). As a full verb or a TA in the present perfect tense, *häst* occurs almost exclusively in the Pomerano data set. In (7), however, the informant detects a problem, probably the missing MV, and therefore produces the verbal unit *ha küüst*. *Ha* represents the expected ‘past tense’ morphology, but does not agree with the 2SG-subject pronoun. *Küüst*, however, is not only a ‘past tense’ form, but also agrees with the subject pronoun.^11^

If we do not accept this as an instance of double finiteness, we have to assume at least three different types of past participles (*küüt*, *küün*, and *küüst*) and curiously, all three could be said to agree with their respective subject pronoun. Moreover, comparable types of double finiteness can be found in Polish and in Low German varieties from Europe.^12^ With (7) and several other translations, it becomes clear that the single ‘group’ 3+-speaker Pom-65 is not a unique exception. Accepting the presence of two finite verbs in (6) and (7), we have to modify question (vi) from Sect. 1. It does not only have to address the lack of a finite verb in finite clauses, but also the presence of two finite verbs in such clauses.

Tokens such as (8a–b), which are produced by 21 group 1-speakers, present, at least at first glance, far less conspicuous translation variants. Here, the finite TA and the past participle of the MV are separated by the subject pronoun *ik* ‘I’.

**Table:** 

stimulus <45>		Portuguese: Ontem eu poderia ter vendido o anel.						
		English: Yesterday I could have sold the ring.						
(8)	a.	Gistern	haar	ik	küüt	dai	fingering	forköipa.
		*yesterday*	*had.1SG.PST*	*I.1SG.NOM*	*can.PP*	*the*	*ring*	*sell.INF*
						(Pom-105; f/37/Port>Pom-64%)		
	b.	Gistern	had	ik	küüt	mijne	fingering	forköft
		*yesterday*	*had.1SG.PST*	*I.1SG.NOM*	*can.PP*	*my*	*ring*	*sold.PP*
		häwa.						
		*have.INF*						
							(Pom-154; f/38/Pom)	

Both the number of verbs and the form of the TA correspond to the morphosyntactic forms that can be found in examples from Tressmann’s (2006) dictionary. In (9) from page 327, we present one of them with our gloss and our English translation. This example resembles (8b), but there are also examples that resemble (8a). The subject pronoun in (9) appears clause-initially, so we cannot tell whether the subject pronoun would separate *haarst* and *müst* in the presence of a clause-initial adverb.^13^ This is also true for most PCF+MVs in the Pomerano data set.

**Table:** 

(9)	Julius	hät	sägt,	duu	haarst	müst	dai	doir
	*Julius*	*has*	*said*	*you.2SG.NOM*	*had.2SG.PST*	*must.PP*	*the*	*door*
	taumåkt	häwa.						
	*closed.PP*	*have.INF*						
	‘Julius said you should have closed the door.’							
							(Pomerano from ES)	

Like (5a–c), (8a–b) show variation in the number of clause-final verbs. Token (8a) with a total of three verbs represents three informants, token (8b) with a total of four verbs 18 informants. Ten of these 18 translations feature the TA with ‘past tense’ morphology and a second past participle. According to our assumption in question (ii), this second past participle is semantically superfluous and thus, these translations could be regarded as cases of overcoding (cf. the discussion in Sect. 5.2.1). Importantly, the clause-final appearance of three verbs, as in (5c), hardly ever coincides with the topological separation of the TA and the MV in (8a–b) (cf. Table 8). This means that the relative positions of the MV *küüt* and the subject pronoun *ik* ‘I’ in (5a–c) and (8a–b) do not only represent a superficial PF-difference in linearization, but are of crucial importance for the morphosyntax of the whole sentence. Only if they surface adjacently are there many instances of clause-final verbal triples, as in (5c), and hardly any ‘past tense’ forms of the TA.

One may thus presume that the question of whether the subject surfaces to the left or to the right of the finite verb (cf. Bjorkman and Zeijlstra 2019, 529–534) or the lack of superficial adjacency of subject pronoun and finite verb (cf. Kaur 2017; Bjorkman and Zeijlstra 2019 for interveners in agreement) constitute the decisive reasons for agreement blocking. *Küüt* in (5a–c), but not in (8a–b), would function as an intervening element. Example (10) demonstrates that this explanation is ill-founded:

**Table:** 

stimulus <39>	Portuguese: A verdade que tu deverias ter dito para o juiz é horrivel.									
	English: The truth which you should have told the judge is horrible.									
(10)	Dai	wårheit,	wats	[0.4]	duu			ha		müst
	*the*	*truth*	*that.2SG*	*[0.4]*	*you.2SG.NOM*			*had.3SG.PST*		*must.PP*
	sägt	häwa	tu	dem	juiz,	[1.3]	is	seir	hässlig.	
	*said.PP*	*have.INF*	*to*	*the*	*judge*	*[1.3]*	*is*	very	ugly	
									(Pom-154; f/38/Pom)	

Token (10) features the relative particle *wats* ‘that’, which agrees with the 2SG-subject pronoun *duu*, a rather frequent phenomenon in the Pomerano data set (cf. Postma 2019, 169–170; Kaufmann and Duran in print). In stark contrast to this, the ‘past tense’ TA *ha* does not agree with *duu* ‘you.2SG’ in spite of the fact that it does not only surface adjacently to *duu*, but also to the right of it. In this example, blocking only occurs with regard to person agreement, but there are innumerous translations of this kind where both ‘tense’ and person agreement are blocked.

A majority of 56 informants are group 4-speakers and produce the following variants:

**Table:** 

stimulus <45>		Portuguese: Ontem eu poderia ter vendido o anel.							
		English: Yesterday I could have sold the ring.							
(11)	a.	Gistern	küün	ik	dai	fingerring	[0.4]	forköft	häwa.
		*yesterday*	*can.1SG.PST*	*I.1SG.NOM*	*the*	*ring*	*[0.4]*	*sold.PP*	*have.INF*
							(Pom-153; m/53/Pom+Port)		
	b.	Am	gistern	küün	ik	dai	fingerring	forköft	
		*at*	*yesterday*	*can.1SG.PST*	*I.1SG.NOM*	*the*	*ring*	*sold.PP*	
		hat	häwa.						
		*had.PP*	*have.INF*						
							(Pom-111; f/28/Pom)		

Once again, we are faced with different numbers of clause-final verbs. Translation (11a) features two clause-final verbs, namely *forköft häwa* ‘sold have’, and covers 55 of the 56 tokens. With one important exception, (11a) conforms to Iatridou’s (2000) assumptions about past counterfactuals. The finite MV *küün* with ‘past tense’ morphology expresses the counterfactual layer, while the past participle *forköft* ‘sold’ expresses the temporal layer. Just like (8b), the unique translation (11b) with two clause-final past participles may be another example of overcoding. The one exception to Iatridou (2000) is that translations (11a–b) feature a finite form of the MV as the highest verb and this could, just like in StG, indicate a case of epistemic rather than dynamic/deontic modality.^14^

Indeed, although an epistemic reading of the Portuguese stimulus version of sentence <45> (or of its English translation) is marked, it is not impossible. However, the fact that identical distributions occur in stimulus sentences that do not allow for an epistemic reading (cf., e.g., stimulus sentence <29>^15^) and the fact that the translations of stimulus sentence <45> in MLG hardly ever feature a finite MV as highest verb^16^ make it clear that the variation in Pomerano is not the result of different interpretations by the informants or of calquing due to the Portuguese finite MV *poderia* ‘could’. Therefore, one can conclude that MVs in Pomerano can take scope over the TA and still indicate dynamic/deontic modality.

The last possible explanation for the variation found in these PCF+MVs that we have to exclude is that translations that feature the construction *hä(t) küüt ik*, as in (5a–c), represent an analytic alternative to synthetic *küün ik*, as in (11a–b) (cf. Postma 2019, 126). This could then be a case of *Präteritumsschwund* ‘atrophy of the preterite’. Granted, in general, the assumption that (5a–c) are the result of an avoidance of a more synthetic morphology makes sense in a variety in which *Präteritumsschwund* is a very frequent phenomenon.^17^*Präteritumsschwund*, however, would not explain why tokens such as (5a–c) are usually accompanied by far-reaching morphosyntactic changes that affect the whole clause. Much less would it explain the frequent occurrences of non-adjacent variants such as (8a–b), in which the TA surfaces with ‘past tense’ morphology.

The last variant is represented by six group 3-speakers. Their translations display the same lack of finiteness as the consequent of (4b). Unlike (4b), however, all six translations feature just two clause-final verbs:

**Table:** 

stimulus <45>	Portuguese: Ontem eu poderia ter vendido o anel.						
	English: Yesterday I could have sold the ring.						
(12)	Gistern	küüt	ik	dai	fingerring	forköft	häwa.
	*yesterday*	*can.PP*	*I.1SG.NOM*	*the*	*ring*	*sold.PP*	*have.INF*
						(Pom-50; f/37/Pom+Port)	

In (12), the nonfinite form *küüt* occurs where we would have expected a finite MV *küün*, as in (11a–b), or a more complex construction such as *hät küüt ik*, as in (5a–c). Interestingly, informant Pom-108, who is responsible for (4b), is a group 4-speaker. In sentence <45>, he produces variant (11a) with a finite MV with ‘past tense’ morphology. As we have already speculated about an erosional sequence *hät* > *häär* > *hä* > *ä* > *ø* in question (v) of Sect. 1, informants such as Pom-108 are of the utmost importance as they show us how the different translation types relate to each other. Blocking of ‘tense’ and person agreement in the TA of (5a–c) may thus be a first step, (complete) phonetic erosion of the TA in (4b) and (12) would then pave the way for tokens such as (11a–b). The precise morphological form of the TA is, therefore, of the utmost importance.

## Variation in PCF+MVs of Pomerano: An instance of language change

As agreement blocking seems to be intimately related to the structural/topological position of the MV, the following sequence of developmental stages for PCF+MVs in Pomerano will be assumed (cf. Fig. 1).

Figure 1 sorts the different groups of speakers into subsequent stages of an instance of language change. For each step, the first line names the respective group. The second line presents the group’s typical variant(s). Finally, the third line specifies the number of possible coding elements for the two layers of PCF+MVs, i.e., the number of verbs with ‘past tense’ morphology and the number of past participles. These indications refer to all PCF+MVs, not just to the tokens of sentence <45>. The initial state of Fig. 1, which resembles the structure of the StG pendant of sentence <45>, does not occur in RS. It does, however, exist as a rare option in ES:

**Table:** 

stimulus <45>	Portuguese: Ontem eu poderia ter vendido o anel.						
	English: Yesterday I could have sold the ring.						
(13)	Gistern	ha	ik	dai	fingering	forköipa	küüt.
	*yesterday*	*had.1SG.PST*	*I.1SG.NOM*	*the*	*ring*	*sell.INF*	*can.PP*
						(Pom-739; m/59/Pom)	

In (13), the TA appears with the expected ‘past tense’ morphology and the MV surfaces clause-finally, in the position that we assume to be its original position. In this case, the ‘past tense’ morphology of the TA expresses the counterfactual layer of PCF+MVs, whereas the clause-final past participle of the MV expresses its temporal layer. There is thus no difference to the coding of PCF-MVs, as in (1). Crucially, none of the few translations with the MV in clause-final position shows any lack of ‘tense’ or person agreement.

Obviously, the question now arises why we sort the extant groups of speakers in RS in the way we do. We will give many (derivational) reasons for this sorting in this section and in Sect. 5. At this point, however, we will focus on the distribution of the different variants and on the average age of the respective informants. As Table 4 is only concerned with sentence <45>, we can add the data from ES and RO, where two more translation variants appear (cf. Group 0 and Group 1+).

**Table 4:**
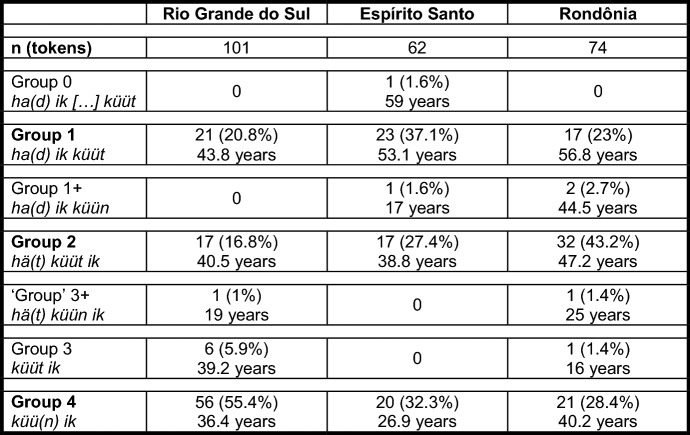
Frequency of and the informants’ average age in seven variants of PCF+MVs in sentence <45> in RS, ES, and RO

Due to the inclusion of the data from ES and RO, one further piece of information had to be changed. *Küün* in these two speech communities frequently surfaces as *küü* without the final [n] (cf. Table 3 and Kaufmann and Duran in print). Likewise, final [t] is sometimes lost, and therefore, it is not clear whether *küü* derives from *küün* or from *küüt*; i.e., it is not possible to clearly distinguish group 3- from group 4-speakers. Translations with *küü* as the highest verb are nevertheless subsumed under group 4 as final [n] is dropped more often than final [t].

We will only comment on the large groups of Table 4 (in bold print), which demonstrate two crucial differences between RS, on the one hand, and ES and RO, on the other hand. First, group 4 is the largest group in RS, but not in ES and RO. There, groups 1 and 2, respectively, are larger. If our assumption that the different variants represent subsequent stages in an instance of language change is correct, this means that the speech community in RS is the most progressive, since it is only there that a majority of informants have already reached the final stage.^18^

Second, in the two less progressive speech communities, the age distribution between the different variants strongly suggests language change, at least in an apparent time scenario. Group 4-speakers in ES and RO are significantly younger than group 2-speakers, who, in turn, are significantly younger than group 1-speakers (ES: *F*(2, 57) = 21.2; *p* < 0.001***/RO: *F*(2, 67) = 5.1; *p* = 0.008**^19^). In RS, the age distribution is less clear. Although the average age also drops for each stage, the differences are not significant. However, things become somewhat clearer when we look at all tokens of PCF+MVs in RS. Those with a TA as highest verb (groups 1, 2, and 3+) are produced by informants who, on average, are 40 years old, while the informants who produce tokens with a MV as highest verb (groups 3 and 4) are 37 years old, a highly significant difference (*F*(1, 1076) = 10.1; *p* = 0.001**).

In comparison to the group 0-variant (cf. (13)), a first consequence of the attraction of the MV to a higher structural position can be detected among group 1-speakers. These speakers’ prototypical feature is *ha(d) ik küüt*, but despite the frequent ‘past tense’ morphology of the TA, they tend to add another past participle, as in (8b). Their PCF+MVs thus feature zero or one verb(s) with ‘past tense’ morphology and one or two, rarely three past participles. In most cases, they possess between one and three elements to code the two levels of past in PCF+MVs. A total of 36 of their 230 tokens (15.7%) only feature an auxiliary with present tense morphology and one past participle. These translations may be either considered underspecified for a PCF+MV, or some informants may have reduced the cognitive complexity of the stimulus sentence by producing a past factual clause with a MV. For sentence <45>, this would mean something like *Yesterday I was able to sell the ring*. Before we continue our discussion of Fig. 1, Table 5 summarizes the type and the morphological form of the highest verb in 963 PCF+MVs from RS.

**Table 5:**
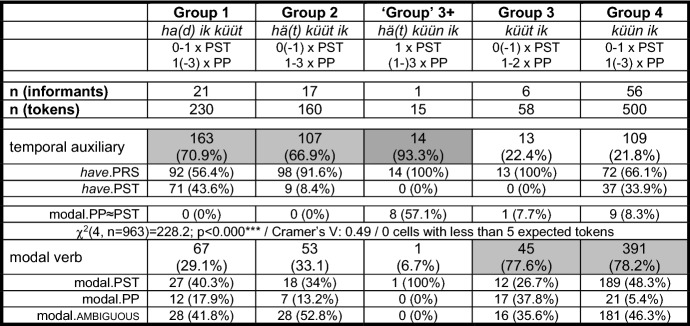
The highest verb in 14 PCF+MVs and in stimulus sentence <9> with an epistemic MV

Table 5 refers to the tokens of the epistemic sentence <9> (cf. (7)) and to all PCF+MVs, with the exception of sentence <45>, the sentence that constitutes the base for the grouping. For the TA, the distribution refers to forms with present tense (have.PRS) or with ‘past tense’ morphology (have.PST) and to MVs in the scope of this auxiliary that appear with ‘past tense’ morphology (modal.PP≈PST; prototypically the ‘group’ 3+-speaker). For the MVs, the distribution refers to ‘past tense’ morphology (modal.PST), to the appearance of a past participle as highest verb (modal.PP; prototypically group 3-speakers), and to unclear cases with *müst* (modal.ambiguous). *Müst* is ambiguous because it can represent both finite forms, i.e., ‘must.SG.PST’, and the non-finite form of the past participle, i.e., ‘must.PP’ (cf. Postma 2019, 111).

The most important information from Table 5 is that the overall distribution of all groups and the internal distribution of the MVs in groups 3 and 4 are, despite some variation, in synchrony with the speakers’ preferences with regard to sentence <45>. This means that the unembedded context of this root clause reveals the informants’ general morphosyntactic behavior in PCF+MVs quite well.

The progression from group 1- to group 2-speakers with the sequence *hä(t) küüt ik* in the CP-domain brings the MV into even closer contact with the TA. This causes an even higher amount of blocking of ‘tense’ and person agreement. While group 1-speakers produce 42.6% (98 of 230 tokens) of verbs with ‘past tense’ morphology (have.PST + modal.PST), this share drops to 16.9% in group 2-speakers (27 of 160 tokens). Group 2-speakers feature zero verbs or, rarely, one verb with ‘past tense’ morphology and between one and three past participles. They have thus between one and four coding possibilities for the two levels of past in PCF+MVs. A total of 37 of their 160 tokens (23.1%) is underspecified with regard to the standard codification of PCF+MVs (cf., e.g., (5a)).

The bifurcation in Fig. 1 from group 2-speakers to group 3-speakers, on the one hand, and to the only ‘group’ 3+-speaker, on the other hand, is marked by further morphosyntactic changes. The translation variant of the ‘group’ 3+-speaker displays double finiteness in *hä(t) küün ik*. His translations always feature one verb with ‘past tense’ morphology (mostly the MV as second-highest verb, once as highest verb) and between one and three past participles. They thus possess between two and four coding possibilities for the two levels of past. Not a single of the 15 tokens is underspecified.

Group 3-speakers frequently lack any sign of finiteness. Their crucial feature in sentence <45> is *küüt ik.* These speakers normally feature zero verbs with ‘past tense’ morphology—only 20.7% (12 of 58 tokens) do so—and produce one or two past participles. They have thus between one and three coding possibilities for the two levels of past. Three of their tokens (5.2%) are underspecified. If the MV is their highest verb, they use the highly marked participial form in 37.8% of the tokens and a finite form in just 26.7% (35.6% of the relevant tokens are ambiguous). The share of non-finite clauses is thus much higher than in any other group.

Before we turn to group 4-speakers, it is worth noting once again that there is a steady, albeit non-significant drop in age from the very top to the very bottom of Fig. 1 (cf. Table 4). Group 1-speakers are, on average, 7.4 years older than group 4-speakers. In order to refine this picture, we can compare the age distribution of tokens of group 2-speakers with TAs. These speakers are crucial because they mark the decisive switch from TAs to MVs as highest verb. By comparing the informants’ age in tokens with *hät*, *häär*, and *(h)ä*, the progressive phonetic reduction of the TA becomes manifest in Table 6.

**Table 6:** Age distribution of three present tense forms of *häwa* in group 2-speakers (1/2/3SG-contexts)

n (token)	n	Age
*hät*	27	48.4
*häär*	22	35.5
*(h)ä*	64	32.5

Table 6 exhibits a highly significant age difference (*F*(2, 110) = 10.2; *p *< 0.001***). The younger the speakers are, the more they use *häär* and *(h)ä* instead of *hät*. This confirms the assumed sequence *hät* > *häär* > *hä* > *ä* > *ø* of question (v) in Sect. 1 and is represented in the connecting line between groups 2 and 3 in Fig. 1. Importantly, no other group displays such an age difference.

The step from group 3- to group 4-speakers in Fig. 1 is characterized by the change from a non-finite to a finite MV as highest verb. Interestingly, only 5.4% of the tokens of group 4-speakers feature a non-finite MV, while 48.3% feature an unambiguous finite MV. Group 4-speakers feature zero or one verb(s) with ‘past tense’ morphology and one, two, and, rarely, three past participles. They have between one and four coding possibilities for the two levels of past. In total, 34 of their 500 tokens (6.8%) are underspecified.

Intriguingly, the configuration of group 4-speakers with regard to the two levels of past coincides with the configuration of group 1-speakers although the coding mechanisms are very different. Likewise, the share of tokens with ‘past tense’ morphology for group 4-speakers is, at 45.2% (226 out of 500 tokens), comparable to the share of group 1-speakers (42.6%), but very different from group 2- and group 3-speakers (16.9% and 20.7%, respectively). These facts suggest that the whole, seemingly confused picture of Fig. 1 is nothing but a sequence of repair operations. In non-technical terms, the intrusive raising of the MV in group 1 and, even more so, in groups 2 and 3 causes the blocking of ‘tense’ and/or person agreement of the TA, which eventually disappears. A first repair mechanism for the loss of ‘past tense’ morphology is the realization of one (sometimes two) additional copy (copies) of the TA which allows for the appearance of one (or two) additional past participle(s).

Though the increase of past participles succeeds in coding both the counterfactual and the temporal layer, the additional verb(s) lead(s) to more and more complex morphosyntactic configurations, the climax of which is (5c). This translation features two verbs in the CP-domain and a clause-final verbal triple. With the (phonetic) disappearance of the TA in the CP-domain, group 3-speakers start to reduce this morphosyntactic complexity by eliminating a semantically inactive TA. This makes the MV the only verb in the CP-domain, an element that first appears as a past participle (group 3) and then with its expected ‘past tense’ morphology (group 4). This last step is again supported by age differences. As there are many group 4-speakers, Table 7 distinguishes four subgroups according to their share of TAs as highest verb in all PCF+MVs. The smaller this share, the more asterisks are added to the subgroup’s name.

**Table 7:**
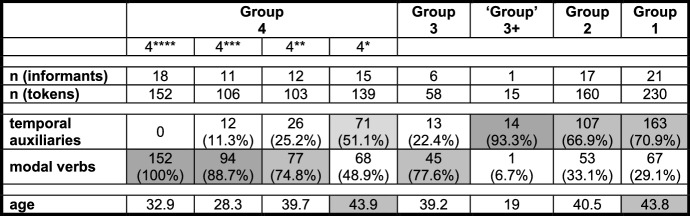
Comparison of eight (sub)groups of speakers

If we compare the age distribution with the relative share of finite TAs, it becomes clear that the finite MV of group 4-speakers does indeed constitute an innovative trait in PCF+MVs. Not only do group 4-speakers represent the (non-significantly) youngest group with an average age of 36.4 years (‘group’ 3+ is not a group), but within this group there is a negative correlation between age and the increasingly dominant use of the MV as highest verb. However, there is only a statistical tendency with regard to the average age of the four subgroups (*F*(3, 52) = 2.7; *p* = 0.056^(^*^)^). Nevertheless, the average age of the group 4-speakers that produce PCF+MVs with a MV as highest verb is 35.2 years, while the average age for tokens with a TA as highest verb is 43.8 years. This difference is highly significant (*F*(1, 498) = 25.4; *p* < 0.001***) and, importantly, no other group exhibits a comparable age difference.

## Derivation and spell-out of PCF+MVs in Pomerano

### Introductory comments

At this point, there can be little doubt that the coding differences in PCF+MVs are linked to the scope rivalry between the TA and the MV. In this section, we will give a more technical account of this rivalry. However, before doing so, we will repeat the six questions from Sect. 1, adding some conclusions from Sects. 3 and 4.

(i) Why does the TA in PCF+MVs frequently appear in its default form (3SG.PRS), i.e., what blocks ‘tense’ and/or person agreement in this clause type?
(ii) Does the blocking of ‘past tense’ morphology in the TA cause the appearance of (an) additional past participle(s) that express(es) one of the two levels of past of PCF+MVs?

Questions (i) and (ii) were dealt with in Sects. 3 and 4. Their derivational background will be discussed in Sect. 5.2.1.

(iii) Does Pomerano really allow two verbs in the CP-domain and what does this mean for the positions of preceding constituents such as *den* ‘then’ or *gistern* ‘yesterday’ and following constituents such as *hai* ‘he’ or *ik* ‘I’?
(iv) If (iii) is answered in the positive, an ensuing question is what the derivational/semantic cause for the two verbs in the CP-domain is.

While question (iii) has not yet been touched upon in any focused way, question (iv) was broached in Sect. 3. Both questions will be conclusively tackled in Sect. 5.4.

(v) Despite the fact that *hä*/*häär*/*hät* only represent phonetic variants of the present tense, the question arises of whether a phonetic erosion process *hät* > *häär* > *hä* > *ä* > *ø* exists.
(vi) How does Pomerano cope with the lack of a finite verb in a finite clause and how can we explain the presence of two finite verbs in some translations?

Question (v) was exhaustively dealt with in Sect. 4. This section also broached question (vi). A more detailed discussion will be given in Sects. 5.2.2 and 5.2.3.

### Derivational accounts for group 2- through group 4-speakers

#### Group 2-speakers

In the following step-by-step derivation, we assume head-final phrases in the IP- and the VP-domain and head-initial phrases in the CP-domain. In order to reduce representational complexity, we will abstract away from vPs. Furthermore, the CP-domain will not yet be split; i.e., the positions of subject pronouns and adverbs will only be analyzed in Sect. 5.4. We assume three VPs, one for the TA (V1P), one for the MV (V2P), and one for the main verb (V3P); i.e., we do not assume that TAs and MVs are base-generated in functional phrases such as AuxP or ModP.^20^ With the exception of some group 4-speakers (cf. (23)), the TA has scope over the dynamic/deontic MV. For the IP-domain, we present ModP, TP, and, unlike Embick and Noyer (2007), AgrSP in syntax proper. Not because of semantic, but because of derivational necessities, one more unspecified functional phrase XP is added (cf. (17) and (19)).

The trees in (14a–b), (15a–b), and (17) detail the derivational facts for (5b), the predominant variant of group 2-speakers. Group 1-speakers will be dealt with in Sect. 5.3. We first repeat (5b) without the Portuguese stimulus and without the informant’s characteristics and then present the initial steps of its derivation.

**Table:** 

stimulus <45>		English: Yesterday I could have sold the ring.						
(5)	b.	Gistern	hä	küüt	ik	mijne	fingerring	[1.0]
		*yesterday*	*has.3SG.PRS*	*can.PP*	*I.1SG.NOM*	*my*	*ring*	*[1.0]*
		forköft	häwa.					
		*sold.PP*	*have.INF*

(14) Stepwise derivation of the lower IP-domain and the VP-domain of token (5b) (group 2-speakers) 
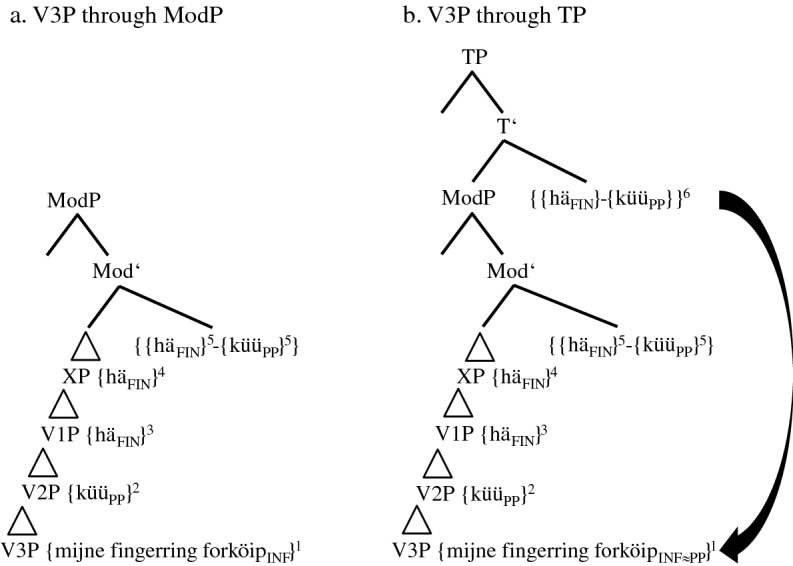

The derivation in (14a) illustrates base generation (not early insertion) and subsequent merges of the verbal roots and the complement of the main verb, i.e., *{mijne fingerring forköip*_*INF*_*}* in V3P, *{küü*_*PP*_*}* in V2P, and *{hä*_*FIN*_*}* in V1P. The subscripts *INF* and *PP* indicate the selectional requirements V2 and V3 have to satisfy. These subscripts can be considered a shorthand for functional phrases not represented. In this, we follow Salzmann (2019, 3): “[…] V1 [in our case *{hä*_*FIN*_*}*] selects an FP into whose head the participle morphology will be inserted.” *FIN* in *{hä*_*FIN*_*}* represents the general finiteness requirements that the highest verb has to satisfy. The timing of merge is indicated by the superscripts 1, 2, and 3.

Step 4 adds the unspecified functional phrase XP (possibly AspP). Due to strict locality, the TA has to move/be copied (in)to the head position of XP. Step 5 concerns the decisive merge of ModP, the head of which possesses a valued and interpretable counterfactual feature (cf. Wurmbrand 2012; Salzmann 2019, 2 for this top-down definition of Agree). The goal *{hä*_*FIN*_*}* would normally value its unvalued counterfactual feature by moving into ModP and thus express the counterfactual layer. After spell-out, this would result in ‘past tense’ morphology. This set of events actually occurs in group 0-speakers (cf. (13)) and in most PCF-MVs (cf. (1)). However, in the PCF+MVs of most informants, both the TA and the MV respond to the probe of ModP. The reason for this multiple agreement (cf. Boeckx 2003) may be that both verbs possess a counterfactual feature, unvalued in the case of the TA and probably valued in the case of the MV (cf. Kulakova and Nieuwland 2016, 61 for this assumption). Be this as it may, both verbs are moved/copied (in)to ModP and with this, their momentous scope rivalry begins.^21^ This double response results in one morphological unit, which we illustrate with a hyphen between the two verbs and an additional pair of curly brackets. Postma (2019, 126) supports this view by writing that “[t]his indicates that V2 [of verb clusters] cannot be a late spellout effect, but a consequence of morphosyntactic incorporation.”

One crucial effect of this incorporation is that, from this position onwards, the TA is blocked and can no longer agree with the heads it passes through. Likewise, the MV partly loses its syntactic independence (cf. Molencki 1998 for a comparable relationship in the history of English). However, this partial loss does not mean that the MV is affixed onto the TA (cf. Tang Boyland 1998 for TAs being affixed to the MV in English past counterfactuals). After all, the eventual winner of the extant scope rivalry is the MV (cf. Fig. 1 and (23)).

As the blocked TA cannot value its counterfactual feature, it cannot express the counterfactual layer anymore. This semantic failure occurs in syntax proper. The lack of ‘past tense’ morphology must, therefore, not be confused with impoverishment in the PF-domain. The past participle *{küü*_*PP*_*}*, which in (13) indicates the temporal layer of the PCF+MV, now has to express the counterfactual layer. Therefore, the valued counterfactual feature of the MV may cause its raising to ModP; the actual job of coding counterfactuality, however, is done by the PP-feature. Semantically, the presence of this counterfactual feature is, just like the subjunctive mood in StG, a mere side effect (cf. Iatridou 2000, 266).

The morphosyntactic consequences of the scope rivalry in ModP becomes even more visible when TP is merged in (14b). Due to its unvalued ‘tense’ feature, *{{hä*_*FIN*_*}-{küü*_*PP*_*}}* is moved/copied (in)to TP.^22^ As the counterfactual layer of PCF+MVs is now expressed by the participial feature of the MV, this feature cannot express the temporal layer anymore. Therefore, a second compensatory strategy is necessary. The probe of TP seems to ‘force’ a participial feature onto the main verb turning *{forköip*_*INF*_*}* into *{forköip*_*INF≈PP*_*}* (cf. the arrow in (14b) and the sign *≈*, which indicates a change in syntax proper). As Embick and Noyer (2007, 304) assume that “the operations that apply at PF are minimal readjustments [...]” and as this long-distance effect results from a semantic necessity, it has to occur in syntax proper.^23^ The merges of the next functional phrases, AgrSP and CP, are represented in (15a–b). In order to save space, we will not repeat the lower parts of IP and the VPs.

(15) Derivation of the upper IP-domain and the CP-domain of token (5b) (group 2-speakers) 
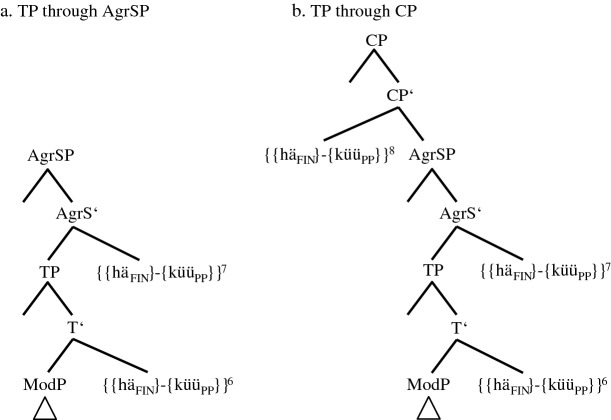

The Φ-features of AgrSP are 1SG. Due to the continuous blocking of *{hä*_*FIN*_*}* inside *{{hä*_*FIN*_*}-{küü*_*PP*_*}}* and due to the fact that the right-hand edge of *{{hä*_*FIN*_*}-{küü*_*PP*_*}}* is non-finite, the unvalued Φ-features of this complex cannot be valued in AgrSP and, therefore, cannot be spelled out (cf. (17)). However, this should not cause a problem because the Φ-features are inside the complex *{{hä*_*FIN*_*}-{küü*_*PP*_*}}* (perhaps in analogy to Bjorkman and Zeijlstra’s 2019, 542 assumption regarding DP-features inside a PP).

The tree in (15a) represents the last verb-relevant merge in a non-root clause. We should, therefore, be able to detect the clause-final sequence V1(TA)-V2(MV) quite frequently. In order to confirm this, (16a–c) demonstrate different ways in which non-root PCF+MVs with three verbs and a TA as highest verb are translated. We have chosen translations with three verbs because the amount of verb (projection) raising is still manageable in this scenario:

**Table:** 

stimulus <57>		Portuguese: O livro que tu deverias ter dado para o teu professor está no teu quarto.									
		English: The book that you should have given to your teacher is in your bedroom.									

(16)	a.	Dat	bauk,	wats	duu	dem	professor	hast			geewa
		*the*	*book*	*that.2SG*	*you.2SG.NOM*	*the*	*teacher*	*had.2SG.PST*			*give.INF*
		mü÷st	oder	dem	leirer	hast	geewa	müst,	is	in	dijne
		*must.PP*	or	the	teacher	had.2SG.PST	give.INF	must.PP	*is*	*in*	*your*
		stuuw.									
		*room*									
								(Pom-133; f/71/Pom+Port)			

	b.	Dat	bauk,	wat	duu	dijm	[0.3]			schaulleirer	geewa
		*the*	*book*	*that*	*you.2SG.NOM*	*your*	*[0.3]*			*teacher*	*give.INF*
		hät	müst,	dat	is	dår	in	dera	stuuw.		
		*has.3SG.PRS*	*must.PP*	it	*is*	there	*in*	*that*	*room*		
									(Pom-128; f/55/Pom)		

	c.	Dat	bauk,	wat	ik	mijm	schaulleirer	hä	müst,		
		*the*	*book*	*that*	*I.1SG.NOM*	*my*	*teacher*	*has.3SG.PRS*	*must.PP*		
		geewa,	dat	is	in	mijne	stuuw.				
		*give.INF*	it	*is*	*in*	*my*	*room*				
									(Pom-58; f/69/Pom+Port)		

The adjacent appearance of V1-V2 in the sequences V3-V1-V2 in (16b) and V1-V2-V3 in (16c) (regardless of the precise position of the object-DP in (16c)) accounts for 96.7% of the 90 relevant tokens. Translations such as (16a), which resemble the StG pendant, are rare. A second confirmation for (15a) comes from the fact that the share of agreeing TAs depends on the verbal sequence in (16a–c). The only sequence in which V1 and V2 are not adjacent and thus the MV cannot possibly block the TA is (16a). Two of the relevant three tokens display ‘tense’ agreement. The respective shares of (16b–c) are significantly lower at 10.3% and 27.1%.^24^ Fortunately, two of the three tokens of (16a) occur with 2SG-subject pronouns where person agreement can be clearly seen. Both display person agreement. Unlike this, the sequence V3-V1-V2 of (16b) shows person agreement in just 42.9% in this context (3 of 7 tokens), while the share is 40% for the sequence V1-V2-V3 of (16c) (2 of 5 tokens).

In view of this, the assumption of a raised MV that blocks ‘tense’ and person agreement of the TA in ModP is not only theoretically possible, but receives strong empirical support. Crucially, ‘tense’ blocking is most common in the sequence V3-V1-V2 (39 tokens). This sequence is, in our view, always the result of head movement of the MV to the TA in ModP.^25^ Unlike this, the sequence V1-V2-V3 (48 tokens) is derivationally ambiguous. It could be the result of head movement of V2 to V1 in ModP and subsequent verb projection raising of V3P to the right of the newly formed unit V1-V2. It could, however, also be the result of cyclic verb projection raising of V2P and V3P to the right of V1. In this case, there should be no blocking effect.

If V1 and V2 form a morphological unit in ModP, which then moves up until AgrSP in non-root clauses (cf. (15a)) and until CP in root clauses (cf. (15b)), its presence in the CP-domain in (5a–c) becomes explainable. This assumption also offers a solution to an important theoretical dispute. Sternefeld (2009, 521) rejects the idea of the formation of complex heads in IP precisely because the resulting complex head is never moved to CP in Continental West Germanic varieties. As this is different in Pomerano, one may either conclude that the sequence V3-V1-V2 and possibly V1-V2-V3 in European varieties of (Swiss) German and Dutch have to be derived in a different way or that these varieties possess a filter that disallows the movement of two verbs into the CP-domain leading to the excorporation of the MV from a complex such as *{{hä*_*FIN*_*}-{küü*_*PP*_*}}*.

The spell-out of (5b) is illustrated in (17). Spelled out constituents appear in bold italics within squared brackets. Spell-out itself is indicated by < for head-initial phrases and by > for head-final phrases. Copies that are not spelled out are double-crossed.

(17) Spell-out of the CP-domain of token (5b) (group 2-speakers) 
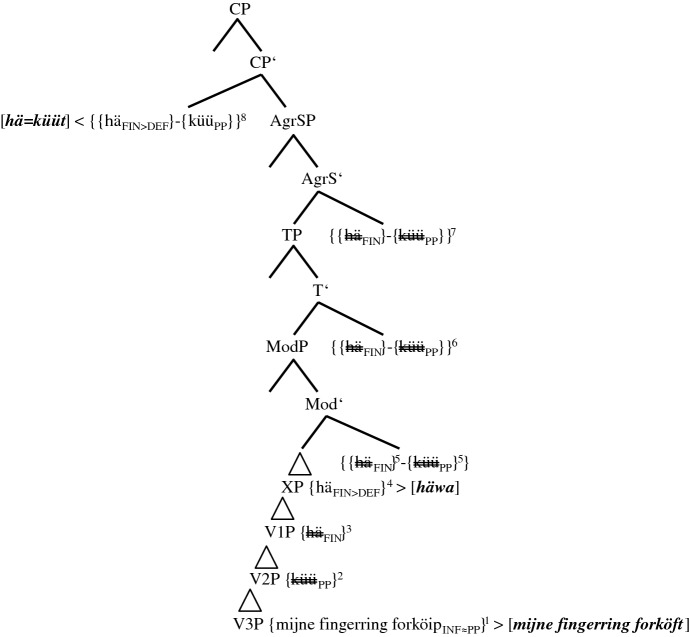

The spell-out of *[hä=küüt]* and *[mijne fingerring forköft]* should be unproblematic. The DM-correlate of the morphological union of *{{hä*_*FIN*_*}-{küü*_*PP*_*}}* is fusion. We illustrate this using the equals sign. Halle and Marantz (1993, 116) write that “[…] fusion takes two terminal nodes that are sisters under a single category node and fuses them into a single terminal node. […] Unlike merger, fusion reduces the number of independent morphemes in a tree.” The TA is spelled out as a phonetically reduced 3SG.PRS-default form *[hä]*. We present the impoverishment of the still unvalued features FIN as FIN>DEF.

The only surprise the reader may feel with regard to (17) could be the spell-out of *[häwa]* in XP. Just like *[hä]*, this copy of *häwa* does not have any semantic impact. Therefore, it must result from a post-syntactic well-formedness condition (cf. Embick and Noyer 2007, 305), solving the selectional mismatch between the MV *[küüt]*, which selects an infinitive, and the past participle *[forköft]*. Localizing *[häwa]* in the PF-domain, we assume that it is not an infinitive regularly selected by the MV *[küüt]*, but a default form without functional morphemes.^26^ The spell-out of *{hä*_*FIN>DEF*_*}* as *[häwa]* in XP is unproblematic since *{hä*_*FIN>DEF*_*}* is not yet blocked by the MV (cf. (14a)).

The basic derivational assumptions in (14a–b), (15a–b), and (17) also cover (5a,c), the two minority options of group 2-speakers. We repeat the two variants before presenting the spell-out of the lower IP-domain and the VP-domain in (18) and (19). The higher regions of these trees are identical to the one of (17) and will, therefore, not be represented.

**Table:** 

stimulus <45>		English: Yesterday I could have sold the ring.						
(5)	a.	Gistern	häär	küüt	ik	mijne	[0.6]	fingerring
		*yesterday*	*has.3SG.PRS*	*can.PP*	*I.1SG.NOM*	*my*	*[0.6]*	*ring*
		forköipa.						
		*sell.INF*						
	c.	Gistern	hät	küüt	ik	dai	fingerring	forköft
		*yesterday*	*has.3SG.PRS*	*can.PP*	*I.1SG.NOM*	*the*	*ring*	*sold.PP*
		hat	häwa.					
		*had.PP*	*have.INF*

(18) Spell-out of the lower IP-domain of token (5a) (group 2-speakers) 
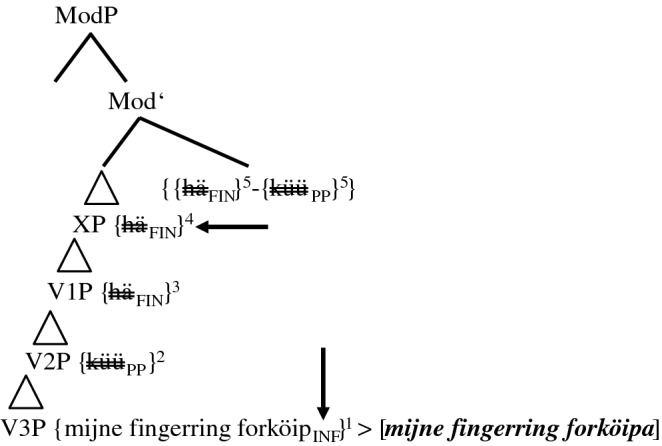

We characterized translations such as (5a) as underspecified, since they feature neither ‘past tense’ morphology of the TA nor a second past participle. Two explanations can be given for this underspecification. (i) The probe of TP is too ‘weak’ to transform *{forköip*_*INF*_*}* into *{forköip*_*INF≈PP*_*}* (cf. the vertical arrow in (18)). If so, a semantic, but not a selectional mismatch results and *{hä*_*FIN*_*}* in XP does not need to be spelled out (cf. the horizontal arrow). (ii) As already mentioned in the discussion of Table 4, some informants may simply reduce the cognitive complexity of the stimulus sentence by translating it as the equivalent of *Yesterday I was able to sell the ring*.

One fact renders this second explanation unconvincing. The predominance of 3SG-default forms *häär* and *hä* in tokens such as (5a) regardless of the person of the subject (pronoun) (cf. Table 6) distinguishes these translations from past factual clauses with or without MVs, in which the TA in the present perfect tense either surfaces as *hät* in 3SG-contexts, as *häst* in 2SG-contexts, or as *häf* in 1SG-contexts. The tree in (19) illustrates the spell-out of (5c).

(19) Spell-out of the lower IP-domain of token (5c) (group 2-speakers) 
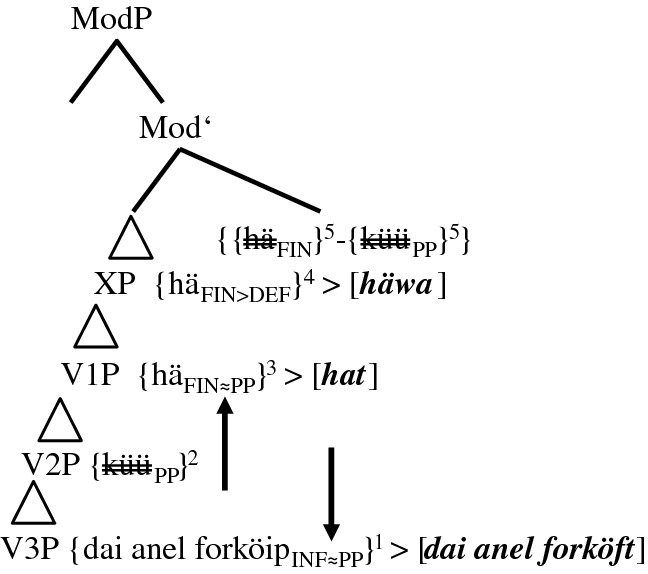

As the probe of TP creates two additional past participles in (5c), a prototypical case of syntactic doubling,^27^ it does not seem to be too ‘weak’, as in (5a), but too ‘strong’. Alongside *{forköip*_*INF≈PP*_*}*, *{hä*_*FIN*_*}* turns into *{hä*_*INF≈PP*_*}* in V1P (cf. the two arrows). Again, the unspecified functional phrase XP spells out as *[häwa]* and satisfies a morphological well-formedness condition of the PF-branch. The reader can now see how fundamental it was to base generate all verbs in the VP-domain. Without multiple copies of the TA that are not yet blocked by the MV, we could not accommodate all forms actually spelled out.

Overlooking the descriptions of Sect. 5.2.1, we are quite sure that we have tried some readers’ patience with expressions such as a probe being too ‘weak’ or too ‘strong’ or a probe ‘forcing’ features onto distant constituents. However, in view of variants such as (5a,c), part of the variation in PCF+MVs in Pomerano is best explained by varying degrees of strength of morphosyntactic mechanisms. Such an assumption may also explain tokens such as (8b) and (9), in which overcoding occurs just like in (5c), as the ‘past tense’ morphology of the TA co-occurs with two past participles.

Intriguingly, there may be a biological parallel to these different degrees of probing. The body’s immune system frequently underreacts, but sometimes also overreacts to diseases such as COVID-19. The overreaction is called the cytokine release syndrome. The mechanism that turns *{forköip*_*INF*_*}* into *{forköip*_*INF≈PP*_*}* in V3P (cf. (14b)) may be likened to the transcription and secretion of a cytokine, while variant (5c) in (19) adds an additional cytokine, namely *{hä*_*INF≈PP*_*}* in V1P, and may be seen as a morphosyntactic equivalent to the overreaction of the immune system.^28^ That such a parallelism is not at all far-fetched can be seen in the following section.

#### The ‘group’ 3+-speaker

‘Group’ 3+ is only represented by informant Pom-65. Outside sentence <45>, however, some translations by other informants, for example (7), share the morphosyntactic characteristics of his token (6).

**Table:** 

stimulus <45>	English: Yesterday I could have sold the ring.						
(6)	Gistern	hät	küün	ik	[0.3]	dai	anel
	*yesterday*	*has.3SG.PRS*	*can.1SG.PST*	*I.1SG.NOM*	*[0.3]*	*the*	*ring*
	forköft	hat	häwa.				
	*sold.PP*	*had.PP*	*have.INF*				

There are three possible coding elements for the two layers of PCF+MVs: two past participles, *forköft* ‘sold’ and *hat* ‘had’, and *küün* ‘can.1SG.PST’, which displays ‘past tense’ morphology. Although (5c) features just as many coding elements, there are two finite verbs in (6), *hät* and *küün*, but only one in (5c), *hät*. The tree in (20), therefore, has to explain how *küüt* in (17) turns into *küün*.

(20) Spell-out of the CP-domain of token (6) (‘group’ 3+-speaker) 
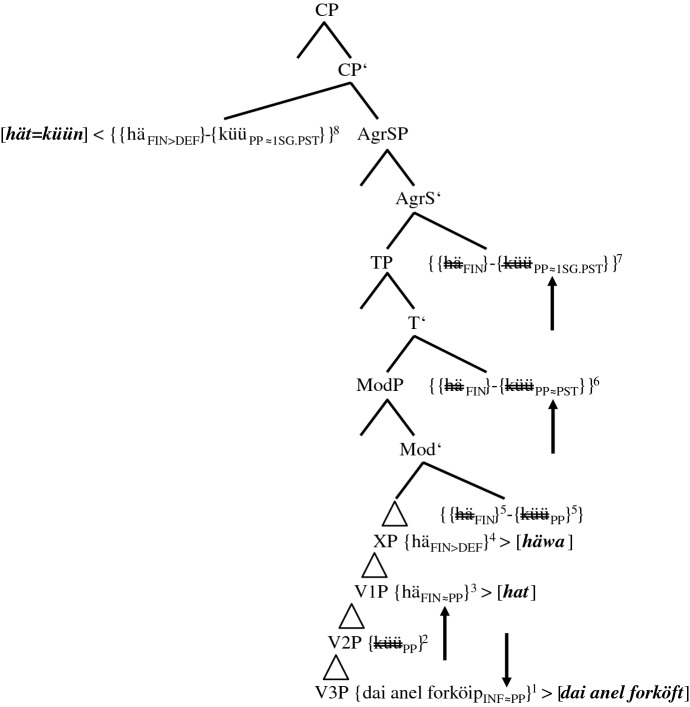

We have qualified the two clause-final past participles in (5c) as a morphosyntactic overreaction to an overly ‘strong’ tense probe since only one additional past participle is needed to code the temporal layer of PCF+MVs. In (20), things escalate even more because we now have to assume that the probe of TP causes three morphosyntactic changes (cf. the three lower arrows). There are two additional past participles, *{hä*_*FIN≈PP*_*}* in V1P and *{forköip*_*INF≈PP*_*}* in V3P, and there is *{küü*_*PP≈PST*_*}* in TP, which will be spelled out as an additional finite verb with ‘past tense’ morphology.

Granted, the ‘past tense’ morphology on the MV does not seem to make a lot of sense semantically since the past participle *{küü*_*PP*_*}* already satisfied the coding necessity of the counterfactual layer. One must not forget though that the TP-probe is a tense probe and this may render ‘past tense’ morphology of the MV a more adequate reaction for some speakers. After all, the presence of the non-finite feature of *{küü*_*PP*_*}* in the IP- and the CP-domain is in itself a conundrum.

Obviously, the consequence of this assumption is that the complex *{{hä*_*FIN*_*}-{küü*_*PP*_*}}* is, unlike in (14a–b), (15a–b), and (17) through (19), not morphologically opaque. This may explain the rather exceptional status of Pom-65. In any case, the morphological activity of *{{hä*_*FIN*_*}-{küü*_*PP≈PST*_*}}* continues, as the MV agrees not only in ‘tense’ but also in person (cf. the arrow in AgrS^0^).^29^

An indication for the rather coherent behavior of the different groups with regard to such overreactions is the distribution of clause-final constructions such as *forköft hat häwa* ‘sold had have’ in (5c) and (6). The following binary logistic regression analysis examines the impact of eight independent variables:

**Categorical variables**

- group of speakers (4 variants; contrasting variant *group 4*): group 4; group 1; group 2; ‘group’ 3+
- clause type (2 variants; contrasting variant *root clause*): root clause; non-root clause
- conceptual gender (cf. Ackerman 2019 for this terminology) (2 variants; contrasting variant *male*): male; female

**Metrical variables**

- age (in years)
- schooling (in years)
- (competence in) Pomerano
- (competence in) StG
- (competence in) Portuguese

Group 3-speakers had to be excluded because they do not produce a single instance of such triples ((4b) was produced by a group 4-, not a group 3-speaker). The model in Table 8 comprises 1004 tokens (47 tokens with a verbal triple). It includes tokens with TAs or MVs as highest verb, but this variable cannot enter the model because it was used for the grouping of the informants in Sect. 3.

**Table 8:**
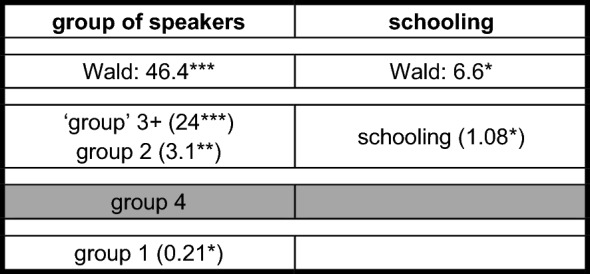
Binary logistic regression analysis for the occurrence of clause-final verbal triples in PCF+MVs

The stepwise forward model selects two of the eight variables and ‘explains’ 16.5% of the variation (Nagelkerkes R-square: 0.165).^30^ The selected variables are shown in the columns of Table 8. Below the indication of the Wald-value, the reader finds the contrastive variant of the categorical variable in the shaded central line. Above the shaded line, the metrical variable and the variants of the categorical variable that increase the probability of clause-final verbal triples are listed with the values of the exponential-function of the regression coefficient β. Below this line, the variant of the categorical variable that decreases the probability of this variant is listed.

The selection of the variable *schooling* is somewhat surprising, but its Wald-value is too low to cause us any discomfort. Four more years of schooling increase the probability of a verbal triple by a factor of 1.36 (1.08^4^). The impact of the speaker group is seven times stronger. The probability for a triple is 24 times higher for the ‘group’ 3+-speaker in comparison to group 4-speakers. Likewise, the probability rises by a factor of 3.1 for group 2-speakers.^31^ Group 1-speakers, however, exhibit a reduced probability of 4.8 (1:0.21) and thus, once more, behave differently from group 2-speakers. The probability of a morphological overreaction of this type thus decreases along the line *‘group’ 3+ > group 2 > group 4 > group 1*. For ‘group’ 3+-, group 2-, and group 1 speakers, this correlates with the structural and superficial distance between the MV and the TA.

#### Group 3-speakers

The central characteristic of group 3-speakers is that their translation variant does not contain a finite verb. We repeat (12) and attach its derivation and spell-out in (21).

**Table:** 

stimulus <45>	English: Yesterday I could have sold the ring.						
(12)	Gistern	küüt	ik	dai	fingerring	forköft	häwa.
	*yesterday*	*can.PP*	*I.1SG.NOM*	*the*	*ring*	*sold.PP*	*have.INF*

(21) Spell-out of the CP-domain of token (12) (group 3-speakers) 
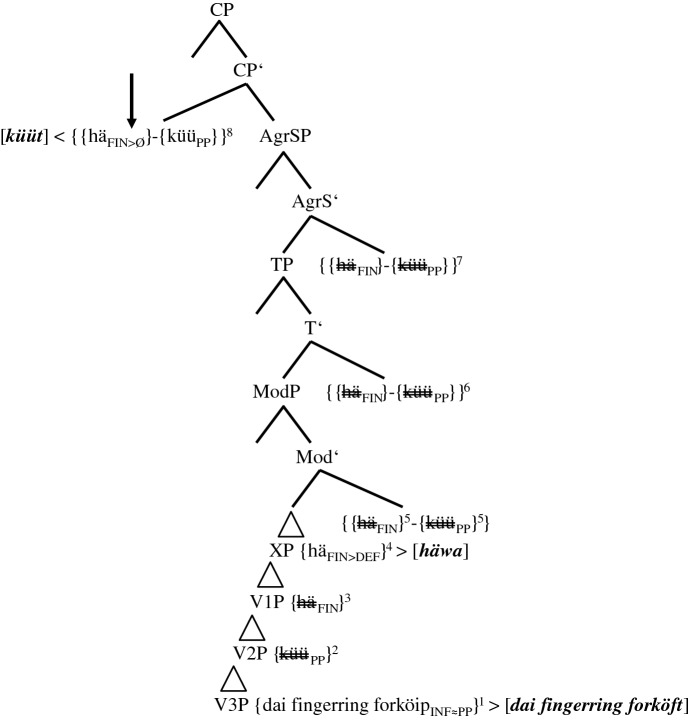

On the one hand, group 3-speakers behave like group 2-speakers, and not like the ‘group’ 3+-speaker, in that the MV *{küü*_*PP*_*}* is spelled out as a past participle. Group 3-speakers thus do not ‘overreact’ to the demands of TP. On the other hand, group 3-speakers are more radical than group 2-speakers in that they suppress the functionless TA by deleting its phonological features, a case of impoverishment. It is at this stage that the MV visibly takes over. An interesting correlation to this is the fact that group 3-speakers do not produce a single token with the clause-final verbal triple (cf. their exclusion in Table 8). One reason for this may be that the multiple spell-out of lower copies of the TA is less probable if the highest copy is not spelled out (but cf. exceptions such as (4b)).

These non-finite clauses may remind the reader of non-root clauses in the (written) German of the 17th, 18th, and 19th century, in which the TAs *haben* ‘have’ and *sein* ‘be’ could be deleted in order to “derank[] subordinate clauses (formally distinguishing them from independent clauses) by expressing fewer finiteness categories or none on the subordinate verb form” (Breitbarth 2005, 46). By comparing the behavior of non-finite clauses in Pomerano to this historical phenomenon, we can check our assumption of the deletion of the TA *hä(t)* empirically.

Table 9 contrasts translations without a finite verb with translations with the TA and *küüt* (excluding tokens with double finiteness). The model comprises 198 tokens (62 tokens with isolated *küüt*) with at least three verbs (in the case of *küüt* as highest verb) or at least four verbs (in the case of the TA as highest verb). With the exception of the variable *group of speakers*, the same independent variables as in Table 8 are applied. Due to the exclusion of the variable *group of speakers*, translations of sentence <45> can now be included. Clauses with *müst* ‘must’ are excluded due to its morphological ambiguity (cf. the discussion of Table 5).

**Table 9:**
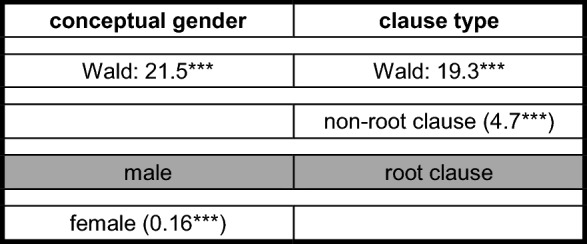
Binary logistic regression analysis for group 2- and group 3-type PCF+MVs

Two of the seven variables are selected and ‘explain’ 29.6% of the variation (Nagelkerkes R-square: 0.296). Crucially, just like in Table 8, competence in Pomerano is not selected; i.e., both phenomena cannot be explained by language attrition. The category female is associated with a reduced probability of isolated *küüt* by a factor of 6.3 (1:0.16). This selection may be related to a lack of overt prestige of the variant in question, but the precise relationship is unclear. In any case, the decisive variable for us is clause type. The probability of the deletion of the TA is 4.7 times larger in non-root clauses than in root clauses. This fits Breitbarth’s (2005, 46) assumption about deranked subordinate clauses. After all, non-root clauses are dependent clauses with a low degree of illocutionary force. In any case, just like non-finite clauses in (written) StG disappeared after some time, the non-finite clauses in Pomerano do not seem to be a robust solution for coding PCF+MVs either. The predominance of group 4-speakers proves this point.

#### Group 4-speakers

We represent the structural configuration of the translation variant(s) of group 4-speakers by means of (11a):

**Table:** 

stimulus <45>		English: Yesterday I could have sold the ring.						
(11)	a.	Gistern	küün	ik	dai	fingerring	[0.4]	forköft
		*yesterday*	*can.1SG.PST*	*I.1SG.NOM*	*the*	*ring*	*[0.4]*	*sold.PP*
		häwa.						
		*have.INF*						

In Fig. 1, group 3-speakers and the one ‘group’ 3+-speaker were vertically localized in between group 2-speakers and group 4-speakers. With regard to morphology, both these groups could represent the link between the two other groups. If it were ‘group’ 3+, one would have to find a mechanism that turned *[hät küün]* into *[küün]*. This could be post-syntactic impoverishment of the phonological features of the functionless TA, a mechanism already applied in group 3-speakers (cf. (21)). However, if ‘group’ 3+ were the precursors of group 4, the dominant group in the Pomerano data set, we would expect more than just one representative.

If group 3-speakers were the structural precursors—and Fig. 1 shows that this is our conclusion—the spelled-out morphology would have to change from *[küüt]* to *[küün]*. This scenario means that after deleting the TA by impoverishment, the isolated non-finite MV has to exchange its non-finite feature for finite features of ‘tense’ and person agreement, a further post-syntactic change. This state of affairs is, among other things, supported by the fact that 12 of 58 tokens (20.7%) produced by group 3-speakers are identical to (11a), while this is only true for one of 15 tokens from the ‘group’ 3+-speaker (6.7%) (cf. Table 5). Moreover, group 4-speakers, just like group 3-speakers, rarely produce clause-final verbal triples, while the ‘group’ 3+-speaker excels in this phenomenon (cf. Table 8). We present the upper parts of the tree that contains the morphological metamorphosis from group 3 to group 4 in (22). The lower parts are identical to (21).

(22) Spell-out of the CP-domain and the upper IP-domain of token (11a) (group 4-speakers before reanalysis) 
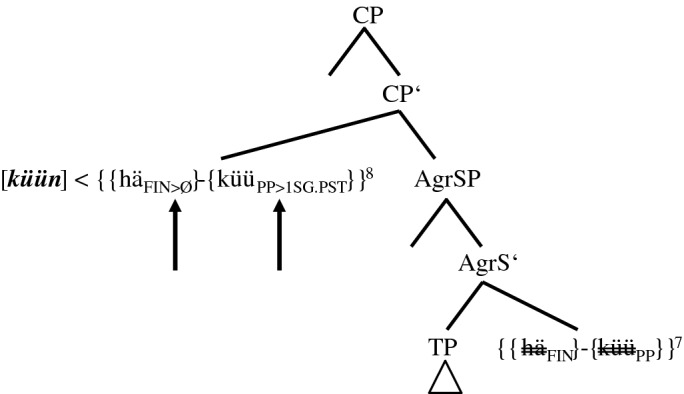

The derivation only differs from (21) in one aspect. Both figures show PF-deletion of *{hä*_*FIN>Ø*_*}* by impoverishment in the CP-domain. The crucial difference is that *{küü*_*PP*_*}* in (22) turns into *{küü*_*PP>1SG.PST*_*}*. Importantly, we do not assume that this change occurs during derivation in TP and AgrSP as in the case of the ‘group’ 3+-speaker (cf. (20)). We rather assume either fission or feature copying, which Embick and Noyer (2007, 309) define in this way: “A feature […] present on a node X in the narrow syntax is copied onto another node Y at PF.” The grammatical reason for this change may be the markedness of verbal non-finiteness in a finite clause.

In any case, (22) only represents derivation and spell-out rules of group 4-speakers that still produce a substantial number of PCF+MVs with a TA as highest verb (group 4*- and group 4**-speakers in Table 7); i.e., PF-deletion of *{hä*_*FIN>Ø*_*}* in these subgroups does not always occur. Actually, the fact that group 4-speakers exhibit ‘past tense’ morphology on the TA quite frequently (cf. Table 5) may indicate that the PF-insertion of this morphology does not only affect the MV, but by analogy also the TA. For group 4***- and group 4****-speakers, who hardly ever produce translations with a TA, we assume that reanalysis has already taken place. These informants base generate the MV in V1P, i.e., above the TA in V2P (cf. (23)).

(23) Spell-out of the CP-domain of token (11a) (group 4-speakers after reanalysis) 
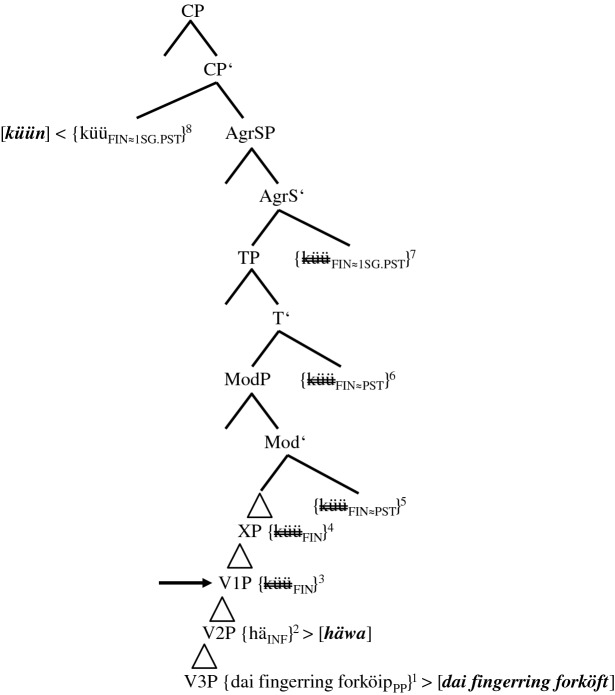

The cognitive reason for this reanalysis is the huge amount of derivational and/or spell-out rules needed in (22). Due to the base generation of the MV in V1P, this verb agrees with ‘past tense’ in ModP and codes for the counterfactual layer of PCF+MVs. As the MV selects the TA *{hä*_*INF*_*}* and as this auxiliary selects a past participle, the temporal layer is also satisfactorily coded for. With this, we have reached the endpoint of Fig. 1. Its initial stage in RS, that of group 1-speakers, will be discussed in the following section.

### A derivational account for group 1-speakers

In (8a), the MV and the TA are separated by the subject pronoun:

**Table:** 

stimulus <45>		English: Yesterday I could have sold the ring.						
(8)	a.	Gistern	haar	ik	küüt	dai	fingering	forköipa.
		*yesterday*	*had.1SG.PST*	*I.1SG.NOM*	*can.PP*	*the*	*ring*	*sell.INF*

For group 2-speakers, we have shown that their translation variants (5a–c) do not contain a verb cluster *stricto sensu* in CP. Instead, the two verbs form one morphological unit in ModP (cf. (14a–b), (15a–b), and (17) through (19)). For group 1-speakers, the decisive question is in which position the MV *küüt* is to be localized. Possible positions include the CP-domain, the base position in V2P (cf. group 0-speakers in (13)), or the IP-domain. For the last two options, one would be compelled to assume that V3P undergoes verb projection raising surfacing to the right of the MV. We will first present our derivation in (24) and then offer empirical support for it.

(24) Spell-out of the CP-domain of token (8a) (group 1-speakers) 
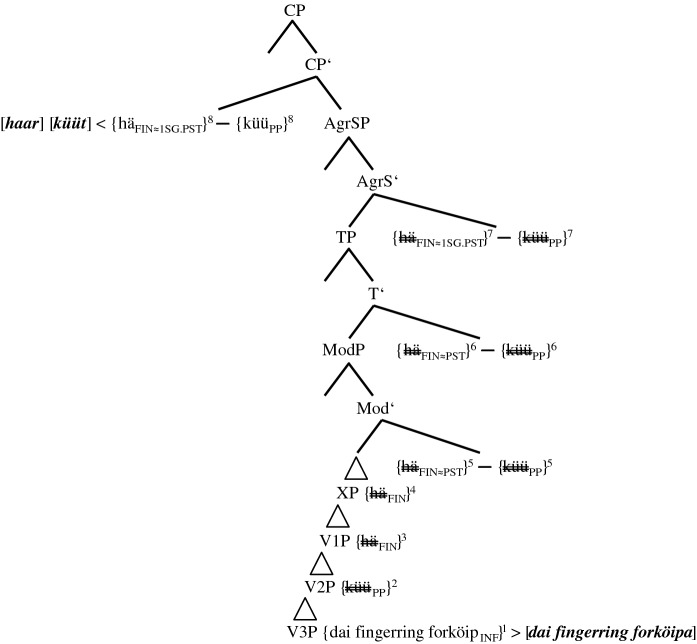

As with group 2-speakers, both the TA and the MV move to ModP. Unlike in their grammar though, the two verbs do not form a morphological unit; they rather adjoin syntactically (hence no additional pair of curly brackets). The PF-correlate of this derivational mechanism is merger, about which Halle and Marantz (1993, 116) write: “[…] merger forms a new word from heads of independent phrases; but these independent heads remain separate morphemes within the new derived word.” Although we are somewhat skeptical with regard to the term “a new word”, the syntactic adjunction of the two verbs is so strong that they move together to TP, AgrSP, and eventually to CP. However, it is not strong enough to always block agreement. Neither is it strong enough to prevent a subject pronoun from surfacing in between.

In (24), *{hä*_*FIN*_*}* first gains ‘past tense’ morphology in ModP and then person morphology in AgrSP. In spite of this, a slight majority of group 1-speakers’ PCF+MVs (54.9%, 101 of 184 tokens; cf. Table 5 including tokens from sentence <45>) display present tense morphology of the TA. So, agreement blocking already occurs in this group, just less frequently than in group 2-speakers (92.7%; 115 of 124 tokens).

In this regard, the intermediate forms, i.e., those with either ‘tense’ or person agreement, are particularly interesting. For a form like *häst* ‘have.2SG.PST’, which displays person, but not ‘tense’ agreement in a PCF+MV, we would either have to assume that the close syntactic adjunction in ModP blocks ‘past tense’ agreement, but is then loosened in AgrSP, or that the extant partial agreement is the consequence of a well-formedness condition on the PF-branch that is, unlike in the case of group 2-speakers, still able to affect the TA. In such cases, Kaur (2017) speaks of a defective intervention that only partially blocks agreement.

As group 1-speakers already display many instances of (partial) agreement blocking, they are fitting precursors of group 2-speakers. This coincides with Halle and Marantz’ (1993, 116) view: “Since both head-to-head movement and merger form structures in which two terminal nodes are sisters under a single category node, both may feed fusion.” Let us therefore explore the CP-domain of PCF+MVs in Pomerano in more detail. In this respect, group 1-speakers are of the utmost importance.

### A derivational account of the CP-domain

As the variation in stimulus sentence <45> was so intriguing, one similar stimulus sentence was added. On the surface, sentence <51> *Onde ele poderia ter pago as dívidas dele?* ‘Where could he have paid his debts?’ does indeed resemble sentence <45>. It starts out with an adverbial element, features a subject pronoun, and contains the MV *poderia* ‘could’. Sentence <51>, however, contains a non-deictic 3SG-subject pronoun and, even more crucially, it is an interrogative, not a declarative clause. We offer three translations that look like the dominant translation variants of group 1 in (25a), group 2 in (25b), and group 4 in (25c):

**Table:** 

stimulus <51>		Portuguese: Onde ele poderia ter pago as dívidas dele?						
		English: Where could he have paid his debts?						
(25)	a.	Wou	ha	hai	küüt	sijn	schuulden	betåld
		*where*	*had.3SG.PST*	*he.3SG.NOM*	*can.PP*	*his*	*debts*	*paid.PP*
		häwa?						
		*have.INF*						
							(Pom-126; f/19/Pom)	
	b.	Wou	häär	küüt	hai	sijn	schuulden	betåld
		*where*	*has.3SG.PRS*	*can.PP*	*he.3SG.NOM*	*his*	*debts*	*paid.PP*
		häwa?						
		*have.INF*						
							(Pom-138; m/47/Port>Pom-68%)	
	c.	Wou	küün	hai	sijn	schuulden	betåld	häwa?
		*where*	*can.3SG.PST*	*he.3SG.NOM*	*his*	*debts*	*paid.PP*	*have.INF*
							(Pom-110; m/35/Pom+Port)	

Table 10 illustrates how the five groups of speakers from sentence <45> translated sentence <51>, when these translations start with the interrogative operator *wou* ‘where’.

**Table 10:**
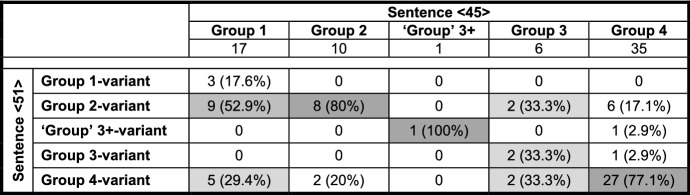
Translation variants of sentence <51> with *wou* ‘where’ for the different groups of speakers

Aside from the expected token of the ‘group’ 3+-speaker, at least three of four informants in groups 2 and 4 display their expected behavior. For group 3-speakers, this share drops to 33.3%. Their other tokens are identical to the translation variants of group 2- or group 4-speakers. As group 3 was seen as a link between these two groups (cf. Fig. 1), this distribution is not too big of a problem. The real exception in Table 10 is group 1. In only 17.6% of their translations do they produce their typical translation variants, namely (8a–b). In more than half of their translations, however, they seem to produce the variants of group 2-speakers.

Our first hunch with regard to this mismatch was to blame the non-deictic nature of the 3SG-subject pronoun in sentence <51>. In line with Sigurðsson’s (2014) assumption about participant linking of 1/2SG-subject pronouns, the “logophoric agent” in sentence <45> may have led to a higher position of deictic *ik* ‘I’. This higher position would then separate the TA and the MV more often than the lower position of *hai* ‘he’.

However, a rather frequent translation problem shows that deixis cannot be the decisive, let alone the sole explanation for the distribution in Table 10. Although stimulus sentence <51> was always presented with a palatalized [dʒ] for /d/ in *onde*, the prestigious variant in Brazilian Portuguese, 14 informants confounded *onde* ‘where’ with non-palatalized *ontem* ‘yesterday’.^32^ Tokens (26a–b) present two such translations. The first token serializes the subject pronoun in between the two verbs, the second one after them:

**Table:** 

stimulus <51>		Portuguese: Onde ele poderia ter pago as dívidas dele?						
		English: Where could he have paid his debts?						
(26)	a.	Gistern	häär	hai	küüt	sijn:	schuulden	betåld häwa.
		*yesterday*	*has.3SG.PRS*	*he.3SG.NOM*	*can.PP*	*his*	*debts*	*paid.PP* *have.INF*
							(Pom-228; f/48/Port>Pom-64%)	
	b.	Gistern	häär	küüt	hai	sijn	schuulden	betåldhäwa.
		*yesterday*	*has.3SG.PRS*	*can.PP*	*he.3SG.NOM*	*his*	*debts*	*paid.PP* *have.INF*
							(Pom-130; m/58/Pom+Port)	

Table 11 compares the relevant translation variants of sentences <45> and <51> distinguishing the different adverbial elements used in sentence <51>.

**Table 11:**
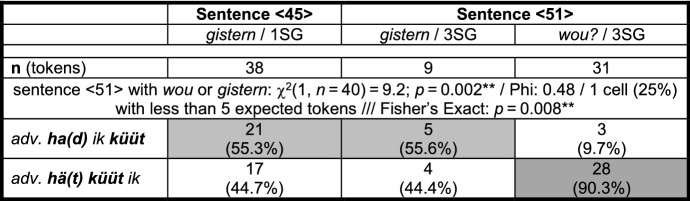
Distribution of group 1- and group 2-type PCF+MVs in stimulus sentences <45> and <51>

When sentence <51> is translated with *gistern*, there is, despite the different deixis of the subject pronoun, no distributional difference whatsoever to sentence <45>. The difference between the tokens of sentence <51> with either *wou* or *gistern*, however, is highly significant. In view of this, the position of the introducing elements *gistern* ‘yesterday’, a temporal adverb, and *wou* ‘where’, an interrogative operator, are decisive. The sequence *wou* ***häär küüt*** *hai* of (25b), frequently occurring in the translations of both group 1- and group 2-speakers, strongly suggests that both verbs are localized in the CP-domain. If this is correct, the two verbs in *gistern ****häär**** hai ****küüt*** in (26a) or *gistern ****haar**** ik ****küüt*** in (8a), the typical translation variants of group 1-speakers, may also be localized in the CP-domain. After all, the adverbial element may have an impact on the eventual position of the TA and the MV in the split CP-domain, but it is hardly conceivable that it interferes with the MV’s necessity to raise to ModP and further. We present the respective derivations and the spell-out of the left periphery of (25b) and (26a) in (27) and (28).

(27) Spell-out of the split CP-domain of token (25b) with an initial interrogative operator (group 1-speakers) 
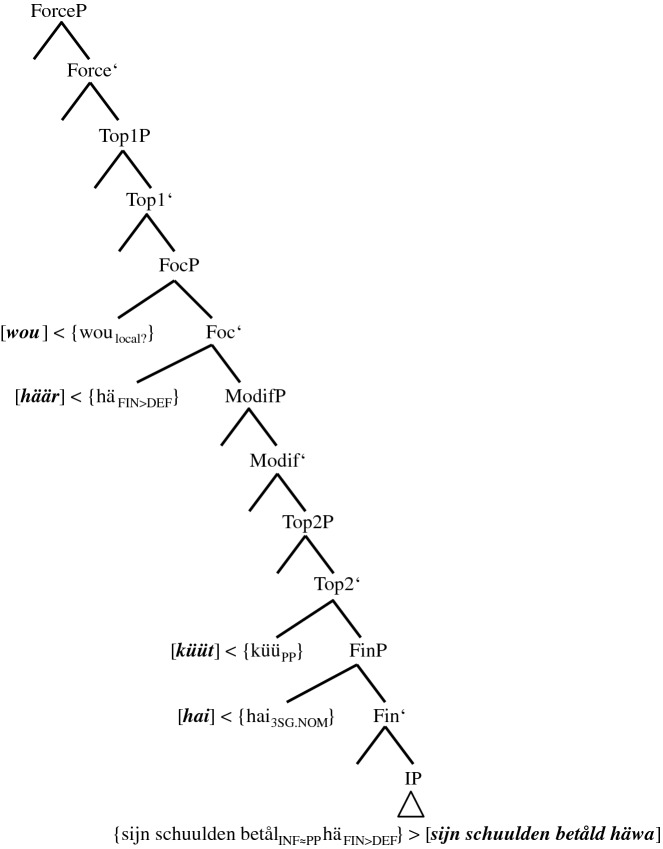

(28) Spell-out of the split CP-domain of token (26a) with an initial temporal adverb (group 1-speakers) 
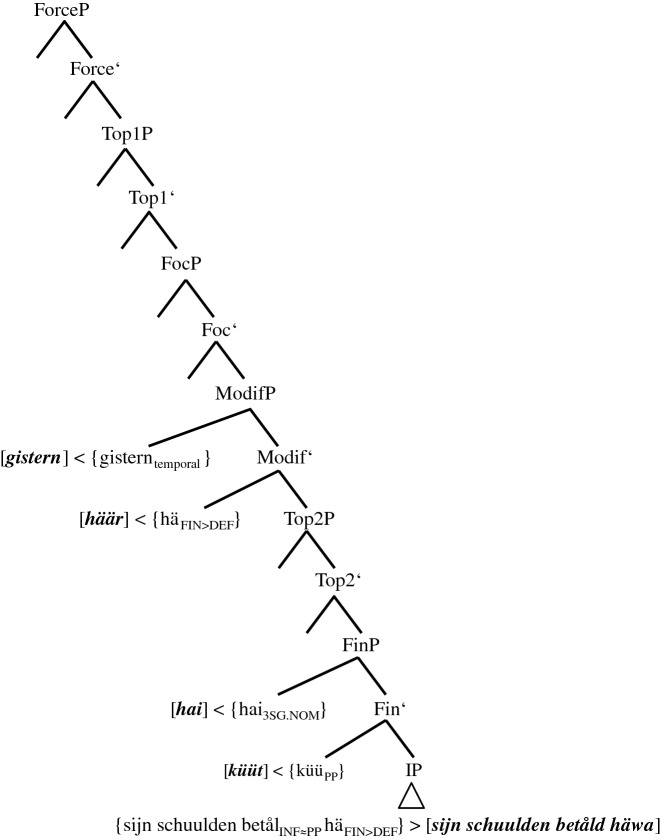

In accordance with Rizzi (2004, 239, 241–242; 1997, 298–299) and Boeckx (2008, 17), the structure in (27) localizes the interrogative operator *{wou*_*LOCAL?*_*}* in Spec/FocP, while the empirical data of Table 11 strongly supports the assumption in (28) that the temporal adverb *{gistern*_*TEMPORAL*_*}* occupies a lower position in Spec/ModifP (cf. Rizzi’s 2004, 241–242 Mod(ifier)P).^33^ Due to the valued operator feature of *{wou*_*LOCAL?*_*}* in FocP and the unvalued operator feature of the morphological or syntactic TA-MV-unit, this unit ends up in (a) higher head position(s) than in translations with *{gistern*_*TEMPORAL*_*}*. As both verbs in (25b) appear to the left of the unstressed subject pronoun *{hai*_*3SG.NOM*_*}*, both are localized in the CP-domain in (27). Although the two verbs in (28) occupy lower positions, there is no reason to assume that they are not localized in the same domain. After all, group 1-speakers produce both translation variants depending on the nature of the clause-initial adverbial element.

If correct, *{hai*_*3SG.NOM*_*}* in between the two verbs in (28) must also be in the CP-domain. With a certain risk of circularity, one may then assume that the subject pronoun in (27) is also in CP. If it raises from Spec/FinP to Spec/Top2P or Spec/ModifP in (27), the minority option for *wou* in (25a) results. The minority option for *gistern* in (26b) results when the MV *{küü*_*PP*_*}* moves to the head position of Top2P in (28). The fact that (28) offers less structural space between the TA *{hä*_*FIN>DEF*_*}* and the subject pronoun *{hai*_*3SG.NOM*_*}* than (27) is the reason for the low frequency of this variant. With this, group 1-speakers can be assumed to produce real syntactic verb clusters in CP and accordingly, we can qualify Abraham’s (1997, 35; cf. also Rizzi 2013, 448) conviction that…

[t]he main tenet of the present essay is that it is difficult, if not impossible, to show that the C=expansion of CP in Rizzi’s sense can be taken to reflect structural properties (distributions, restrictions) that bear on German in any interesting fashion. The conclusion will be that no such expansion is warranted for German and Dutch […]

…by adding “that it is, however, warranted for Pomerano.” The description of the CP-domain of the other groups can be summarized quickly. At least after spell-out (impoverishment of *{hä*_*FIN>Ø*_*}*), group 3- and group 4*^(^*^)^-speakers feature just one verb in the CP-domain. As the constructions *{{hä*_*FIN>DEF*_*}-{küü*_*PP*_*}}* of group 2-speakers (cf. (14a–b), (15a–b), and (17)) and *{{hä*_*FIN>DEF*_*}-{küü*_*PP≈1SG.PST*_*}}* of the single ‘group’ 3+-speaker (cf. (20)) are morphological units, they also occupy just one head position in CP. Therefore, these units should be called morphological rather than syntactic verb clusters. In all these cases, the adverbial element and probably the subject pronoun are also localized in the CP-domain. However, we do not assume this for group 4***^(^*^)^-speakers, who have reanalyzed PCF+MVs (cf. (23)). In this case, we localize non-initial subject pronouns in the IP-domain, though we are not yet able to offer conclusive empirical support thereof. Importantly, the localization for non-pronominal subjects is very different, as (29) and (30) demonstrate:

**Table:** 

stimulus <50>	Portuguese: A que horas tua mãe deveria ter chegado ontem?							
	English: At what time should your mother have come back yesterday?							
(29)	Wat	for-n	stuun	hät	müst	mijn	mama	
	*what*	*for-an*	*hour*	*has.3SG.PRS*	*must.PP*	*my*	*mother.3SG.NOM*	
	gistern	t-huus	kooma?					
	*yesterday*	*to-home*	*come.INF*					
						(Pom-113; m/53/Pom)		
stimulus <53>	Portuguese: Em 1950, o Brasil deveria ter sido campeão do mundo.							
	English: In 1950 Brazil should have become world champion.							
(30)	In	1950	hä	müst	Brasil	campeão	do	mundo
	*in*	*1950*	*has.3SG.PRS*	*must.PP*	*Brazil.3SG.NOM*	*champion*	*of*	*world*
	woura	[0.4]	sin.					
	*become.PP*	*[0.4]*	*be.INF*					
							(Pom-200; f/19/Pom)	

In stimulus sentences with non-pronominal subjects, not a single one of the 33 relevant tokens features sequences such as *wat for’n stuun ****hät**** mijn mama ****müst*** or *in 1950 ****hä**** Brasil ****müst***. The two verbs cannot be separated by non-pronominal subjects. There thus seems to be an upper limit of phonetic material that the left periphery of Pomerano can sustain. If an adverbial and two verbs are already localized there, the CP-domain can only host unstressed subject pronouns, but not non-pronominal subjects.^34^

## Concluding remarks

The focus of this paper was twofold. On the one hand, it empirically documented the variation in the coding of PCF+MVs in Pomerano. On the other hand, it offered derivational explanations for an intriguing change in the morphosyntactic coding of this clause type. As a guideline for analysis, six questions were formulated in Sect. 1. The answers to these questions are intimately related to the behavior of the MV. Eventually, this verb takes scope over the TA, both superficially and structurally (cf. the reanalysis of group 4***^(^*^)^-speakers in (23)). Before this, however, the scope rivalry triggered by the raising of the MV to ModP causes a great deal of morphosyntactic and semantic ‘distress’ (cf. (14a–b), (15a–b), and (17)). By syntactically adjoining to the TA (group 1) or by morphologically uniting with it (group 2), the auxiliary is sometimes/always morphologically blocked (answer to question (i)) and thus loses the capability to code the counterfactual layer of PCF+MVs (cf. Sects. 5.2.1 and 5.3). This functional loss is accompanied by a gradual loss of phonetic weight. Especially interesting for a theory of impoverishment is the fact that the disappearance of the TA in group 2-speakers does not occur categorically, but gradually. This was demonstrated by the age-dependent erosion sequence *hät* > *häär* > *hä* > *ä* > *ø* (answer to question (v); cf. Table 6).

The main reason for the raising of the MV seems to be the fact that it is semantically a better coder for counterfactuality than a TA. However, as long as the MV has not yet gained scope, the scope rivalry with the TA leads to shared movements in the clausal structure of Pomerano (answer to question (iv)) and to two verbs in the CP-domain of root clauses (answer to question (iii); cf. Sect. 5.4). The major semantic consequence of the ‘tense’ blocking of the TA is the necessity of the MV to code the counterfactual layer of PCF+MVs by means of its participial feature. Due to this, it cannot code the temporal layer anymore. In PCF-MVs, this layer is coded by the main verb, which is selected by the TA and appears as a past participle (cf. (1)). In PCF+MVs, the main verb would normally appear as an infinitive selected by the MV. In order to code the temporal layer, it has to be transformed into a past participle (answer to question (ii)). This causes a selectional mismatch between the MV and the main verb, which is solved by the PF-insertion of a second copy of the TA (cf. (17)).

Crucially, we assume that a probe may be too ‘weak’ or too ‘strong’ (comparing this graduality to different degrees of reactions of the immune system at the end of Sect. 5.2.1). Although this gradual concept of probing constitutes the most daring assumption of this paper, it does help us explain why many informants do not succeed at turning the main verb into a past participle despite ‘past tense’ blocking of the TA (too ‘weak’ probing in (18)) or why there is a robust number of translations with clause-final verbal triples and why there are translations with double finiteness (too ‘strong’ probing in (19) and (20)). We may even relate the very fact of two verbs raising simultaneously to ModP as a consequence of too strong a probe in this phrase. With this gradual concept of probing, we have answered the first part of question (vi), the double finiteness in some PCF+MVs. Its second part, the lack of finiteness in some PCF+MVs, was explained by impoverishment of the phonological features of the functionless TA (cf. (21)). This marked state of affairs is eventually resolved by turning the non-finite MV into a finite MV (cf. (22)). Eventually, this leads to the reanalysis of group 4***(*)-speakers (cf. (23)), who base-generate the MV in V1P, above the TA in V2P. These speakers are the youngest ones and may be said to offer the ‘best’ solution in the struggle for scopal dominance (cf. Table 7).

The question of what makes their variant the best one is not hard to answer. On the one hand, tokens such as (11a) do not feature two verbs in the CP-domain; on the other hand, they do not suffer from double finiteness or no finiteness at all. Their MVs simply surface with the expected ‘past tense’ morphology. Due to this, they are also less prone to creating semantically empty copies of the TA (cf. Table 8). Reduction of complexity is thus decisive. Haider (2015, 230) explains this in the following terms:

The selector is blind. Any feature of a grammar that makes grammar acquisition, reception and production easier than a competing grammar will win because brains will acquire this grammar more easily than the less efficient competing grammars, and in the end the winner takes them all.^35^

With regard to language acquisition, one fascinating issue is that although all informants experience all variants in their daily routine (cf. fn. 9), most of them end up with a clear preference for one variant (cf. Table 5). This is a formidable challenge for any theory of language acquisition, in particular for usage-based approaches, but also for the Principles-and-Parameters approach (cf. Boeckx 2008, 7–10 for problems of this approach). Furthermore, the structural insights gained in Sect. 5 should be applied to other languages. It would, for example, be interesting to analyze in detail how English MVs gained scope over TAs in PCF+MVs. English displayed hardly any perfects under MVs before 1350 (cf. McFadden and Alexiadou 2005, 274).

With regard to further research on PCF+MVs in Pomerano, it is important to take an even more detailed look at particular sentences and, crucially, to include the data from ES and RO. Aside from this, an in-depth comparison to past factuals with MVs is necessary. This clause type displays some identical coding strategies—especially with the underspecified variant (5a) though with no blocking of person agreement as in this variant—but it also features marked differences in terms of the position and the number of verbs.

Methodologically, the most important asset of this paper is the language-based grouping of the informants (cf. Sect. 3). For MLG, the explanatory power of this procedure has been demonstrated in Kaufmann (2007) and Kaufmann (2015); here, the analyses of Sects. 4 and 5 bear witness to this power. A second important methodological asset is that the morphosyntactic interrelationships in Pomerano could only be unearthed with data that demonstrate the whole array of variation in a controlled setting. Data elicitation in a multilingual setting by means of translations has thus turned out to provide a valid basis for the successful analysis of a particularly intriguing instance of language variation and change. Therefore, it could and should be used for the analysis of other morphosyntactic phenomena in other multilingual settings.

## Footnotes

1 The orthographic representation of the translations follows Tressmann (2006). In order to improve readability, we use punctuation as in Standard German (StG); i.e., commas separate clauses. The stimulus sentences are always provided in Portuguese and English. In the translations, (un)filled pauses are indicated in brackets (e.g., *[0.3]* or *[äh]*, in the glosses *[eh]*). Break-offs, repairs, and contractions are marked with a hyphen, while a colon represents a markedly prolonged segment. In the glosses, the following abbreviations are used: SG ‘singular’; PL ‘plural’; NOM ‘nominative’; FEM ‘feminine’; PRS ‘present tense’; PST ‘past tense’; SUBJ ‘subjunctive’; PP ‘past participle’; INF ‘infinitive’; IPP ‘*infinitivus pro participio*’; PRT ‘modal particle’; FIN ‘finiteness features’; DEF ‘default form’. Underlined elements represent semantic deviations from the stimulus sentence; a *ø* represents an element that was not translated. Elements that the informants produced although they did not appear in the stimulus are crossed out. Additional verbs are not crossed out in past counterfactuals, in cases of ‘*do*-support’, and in the present perfect tense.

2 Aside from the informant’s coding number, his/her conceptual gender (m=male; f=female) (cf. Ackerman 2019 for this terminology), his/her age in years, and his/her language competence(s) are indicated. If Pomerano is the informant’s dominant language, the label *Pom* is used. Ambilingual informants are represented by *Pom+Port*. If Portuguese is the dominant language, the label used is *Port>Pom*. The actual level of knowledge of Pomerano is then further specified. Pom-53 of (5b), for example, evaluates his competence in Pomerano with 12 out of 14 possible points. This leads to the label *Port>Pom-86%* (cf. Kaufmann 1997, 135–138 for a detailed account of this coding process).

3 Kulakova and Nieuwland (2016, 49–50) write: “Counterfactual thought is thus considered to be a highly complex cognitive capability that develops relatively late in childhood [...] and that is often impaired along with other cognitive functions in clinical conditions like autism, depression, Parkinson and schizophrenia [...].”

4 Grewendorf (1995, 83) describes the semantic impact of the past participle in the present perfect tense: “The compositional meaning of the present perfect tense results from the morphological complexity of this tense that is represented by the aggregate of the finite auxiliary plus the past participle. The meaning of this complex is thus composed by the present tense meaning of the auxiliary and the meaning of the participle. Unlike Zeller (1994), I do not assume that the participle expresses precedence. Following Zagona (1991), I rather assume that the past participle expresses the perfectivity of a(n) event/state. The aspect of precedence is then the logical consequence of the aspect of perfectivity” [our translation]. McFadden and Alexiadou (2005, 276) also consider the past participle decisive for the temporal layer in the resultative configuration *be* + past participle. For *have* + past participle in a past counterfactual context, they consider the TA itself decisive. With this, *had* would code both the counterfactual layer (its ‘past tense’ morphology) and the temporal layer (its quality as auxiliar, i.e., most probably its semantic features). As it is precisely the TA that suffers phonetical and functional reduction in Pomerano, and also in English (cf. Tang Boyland 1998), we find this assumption somewhat problematic.

5 Postma’s (2019, 96–97) discussion about the two forms of *then* in Pomerano from Espírito Santo (ES) confirms this assumption: “The adverb *dun* ‘then’ (Dutch *toen*, Frisian *doe*) has a past feature and combines only with past tenses […] or perfect tenses with a past reading […]. *Dun* contrasts with *den* ‘then’ (Dutch/Frisian *dan*), which has a successive reading and combines with present and future tenses […].”

6 As is the case for almost all past participles in Pomerano, not just those of prefixed verbs such as *unnersreewa* ‘signed.PP’ in (1), *måkt* ‘made.PP’ is not marked by the participial prefix *ge-* (one exception to this is *geeta*, ‘eaten’).

7 *Had* coincides with the form of the past participle *hat* as the final /d/ is devoiced. It is either a non-rhotasized but devoiced form of a historic form *hadde* (cf. Postma 2019, 44) or an analogical formation to present tense *hät*. In the latter case, it should be written as *hat*.

8 Intriguingly, the same verbal triple sometimes occurs in English past counterfactuals (cf. Tang Boyland’s 1998, 5, example (14c): *I would ****have had done**** a ten times better job if...*).

9 Fifty-four women and fifty men were interviewed in RS. The informants are between 13 and 72 years old and live in several settlements in the municipalities of São Lourenço do Sul and Canguçu, two cities that lie 200 and 270 km south of Porto Alegre, the capital of RS. The data were elicited in 2017 and 2018. The data from ES and RO were elicited in 2018 and 2019.

10 Most hesitation phenomena in the translations, especially unfilled pauses, are caused by problems in lexical access and retrieval. The informants frequently tried to produce words in Pomerano for concepts they normally express with a borrowed word from Portuguese (cf. Kaufmann 2017).

11 This constellation may be what Salzmann (2019, 45) has in mind, but does not find in German: “Finite verbal morphology, on the other hand, is (almost) never displaced in German and its varieties. Given the logic of my proposal one might expect this to be possible in ascending orders so that the finite morphology would end up on V2 rather than on V1.” A decisive difference to Salzmann (2019) is that V1 in (7), the TA, does not appear as a default infinitive.

12 Bock (1933, 93) presents relevant PCF+MVs from the border region of Germany with Denmark (thanks to Nobuharu Kakuchi for this hint). In most of his cases, however, both the higher MV (in Pomerano the lower verb) and the lower TA (in Pomerano the higher verb) show the expected ‘past tense’ morphology and agree with the 2SG-subject pronoun. His example (2) is presented as (i) (our gloss and translation): **Table:**

(i)	Dad	šus	dū	nix	hɑ̥̄s	dǭn.
	*that*	*should**.2SG.PST*	*you**.2SG.NOM*	*not*	*had**.2SG.PST*	*done**.PP*
	‘You should not have done this.’					

Bock (1933, 94) writes that “at times one hears the fixed form of *hɑ̥̄ɑ̥̄* for all persons and for both numbers of the preterite” [our translation]. This lack of person agreement could also be seen as a case of blocked agreement. Polish demonstrates similar cases of double finiteness in PCF+MVs. The translation (with thanks to Natalia Boltz) of stimulus sentence <57> *The book that you should have given to your teacher is in your bedroom* (cf. (16a–c)) in (ii) assumes a female listener. There are two finite verbs, the MV *powinnaś* ‘shall.2SG.FEM.PRS’ with present tense morphology (no past tense form of this MV exists) and the TA *była* ‘was.3SG.FEM.PST’ with past tense morphology. Intriguingly, *była* agrees in tense and gender, but not in person.

**Table:**

(ii)	Książka	którą	powinnaś	była	dać	twojemu	nauczycielowi	jest	w
	*book*	*that*	*shall**.2SG.FEM.PRS*	*was**.3SG.FEM.PST*	*give**.INF*	*your*	*teacher*	*is*	*in*
	twoim	pokoju.							
	*your*	*room*							

13 Postma (2019, 110, example (77)) offers a less ambiguous PCF+MVs. In his interrogative example, both verbs are unambiguously adjacent and appear, unlike in (9), with present tense morphology. We present this token with Postma’s translation and a slightly adapted gloss:

**Table:**

(i)	Hät	mücht	hai	dat	trecht	måkt	häwa?
	*has**.3SG.PRS*	*may**.PP*	*he**.3SG.NOM*	*that*	*ready*	*made*	*have**.INF*
	‘Should he have finished it?’						

14 Zagona (2013, 782; cf. also Boye 2016, 132) states: “Although languages vary in the syntax of their modal morphemes, there appears to be a cross-linguistic generalization that epistemic modals are always outside the scope of the clausal event time, while deontic modals need not be.”

15 Stimulus sentence <29> reads *Ele está bravo porque ele poderia ter comprado a casa por muito menos* ‘He is angry, because he could have bought the house for much cheaper’. A majority of 51 translations features a finite MV just like (11a–b) (63.8% of 80 relevant tokens; the figure for sentence <45> is comparable with 58.9%/56 out of 95 finite tokens).

16 Among the 52 relevant tokens of the Mennonite informants, only two (3.8%) contain a MV as highest verb in sentence <45>. If an epistemic reading or calquing were the decisive factor for (11a–b), we would expect a much higher share.

17 Postma (2019) assumes partial *Präteritumsschwund* for Pomerano from ES. He (2019, 104) writes that “[t]he preterite tense of strong verbs is in full use, in contrast to those of weak verbs, which is virtually obsolete […].” The situation in RS seems to be different. Neither in the translations nor in many hours of free conversations do we find many ‘past tense’ forms, except for copula verbs, TAs, MVs, the preterite present verb *waita* ‘know’ and *kooma* ‘come’.

18 In this respect, it is indeed a curious fact that all three Pomeranian speech communities produce the same variants in spite of the facts that a European origin of these variants seems improbable and that until quite recently these speech communities had hardly any contact with each other. This invites speculations about a structural drift as part of the grammatical evolution of Pomerano in Brazil.

19 One asterisk * means that SPSS calculates the probability for a Type I-error between 1% and 5% (0.01 ≤ *p* < 0.05), two asterisks ** that the probability is smaller than 1% (0.001 ≤ *p* < 0.01), and three asterisks *** that it is close to 0% (*p* < 0.001). One asterisk in brackets ^(^*^)^ indicates a statistical tendency with an error margin of 5% to 10% (0.05 ≤ *p* < 0.1).

20 In base-generating the TAs and MVs in VPs, we follow Rothstein’s (2008) assumptions for Swedish. With regard to MVs, he (2008, 102) writes that “[o]ne argument for base generating modal verbs in V_Mod_ is their multiple occurrence within the same TP.” According to Postma (2019, 142), MVs in Pomerano “act as full verbs in forming participles, but most lack infinitival forms […].” MVs in Pomerano are thus partly defective, in between English and Swedish MVs, so to speak.

21 Obviously, some technical issues arise here. As the topological sequence MV-TA never occurs in the CP-domain, the movement of the two verbs does not seem to occur simultaneously; rather, the TA seems to move first. Aside from this, if both the counterfactual feature of ModP and of the MV are valued, the motivation for the movement of the MV is unclear. One could either assume an overactive probe or an overactive goal in the sense of the discussion at the end of this section. Finally, it is unclear whether the MV can move directly from V2P to ModP thus not obeying strict locality (head movement constraint). The tree in (17) suggests such a scenario, as the copy of the TA in XP can be spelled out on its own.

22 We assume that the unvalued features of the TA are still visible, but cannot agree anymore. Visibility could be explained by the TA projecting its features to the morphological unit *{{hä*_*FIN*_*}-{küü*_*PP*_*}}*.

23 Obviously, one will have to harmonize this ‘intrusion’ with phase theory. If we assumed that vP and CP are the only phases that are spelled out, we would have to conclude that *{forköip*_*INF*_*}* has moved above vP, since it could not be affected by TP otherwise. In view of the complications implied in such a solution, our data rather supports an assumption presented in den Dikken and Lahne (2013, 679) according to which “phases are not fixed entities, but dynamic in the sense that phasal domains are extendable (i.e., Spell-out can be delayed) under certain conditions.”

24 The precise specifications are: χ^2^(2, *n*  =  90)  =  7.5; *p*  =  0.023*/Cramer’s V: 0.29/2 cells (33.3%) with less than 5 expected tokens.

25 In spite of the possibility to derive the sequence V3-V1-V2 by verb projection raising (cf., e.g., Wurmbrand 2017; Kaufmann 2007, 189, fn. 32), neither the Pomerano data set nor the MLG data set support such an analysis. The sequence V3-V1-V2 in MLG, for example, is—without a single exception—produced by informants that prefer the unraised sequence V2-V1 in non-root clauses with two verbs (cf. Kaufmann 2007, 185–193, but also Weiß and Schwalm 2017 for a different view). For Eastern Pomeranian varieties in Europe, Weber (2017, 121) also offers a head movement analysis for this sequence.

26 Salzmann (2019, 39) writes in this regard: “Under a pre-syntactic approach, there is absolutely no motivation for these forms, while the supines and the IPP-forms follow naturally under the post-syntactic perspective since they are a default that only kicks in if a verb fails to be associated with functional morphemes.”

27 Barbiers (2013, 6) defines syntactic doubling: “A morphosyntactic feature, morpheme, word or phrase is redundantly expressed two or more times within a sentence.”

28 Fitch (2010, 102) recognizes comparable similarities (bold print of the original dropped): “The existence of a parallel between linguistic hierarchy and the genetic code was recognized almost immediately as the nature of DNA became clear (Monod 1971), and has more recently been dubbed the particulated principle of self-diversifying systems […].” Compare also similar assumptions in Boeckx (2008), who mentions the Fibonacci series on page 118, fn. 60 and, on page 63, states that “[s]yntax creates ever-larger molecules by combining featural atoms through iterated use of *Merge*.”

29 A technical solution to this state of affairs could be that the MV and not the TA projects in ModP (cf. fn. 22). As the MV is more prominent in such a constellation, it could agree more easily with the probes of TP and AgrSP. Abstracting away from the term “‘extra’ grammatical operation”, the finiteness of the MV in (6) may also be explained in the terms of Obata and Epstein (2011). With regard to *whom*, they (2011, 131, fn. 19) write: “One might wonder how the current system deals with a sentence like *Whom did you see?* in that the topmost copy of *whom* has neither [Case] nor [ϕ], which are already split off in the course of the derivation, but still exhibits the Case morpheme -*m*. With respect to this problem, one might adopt Lasnik and Sobin’s (2000) analysis, where the addition of the morpheme -*m* is an independent operation of Case valuation. That is, -*m* is not an accusative Case morpheme accompanying Case valuation but rather is attached by an ‘extra’ grammatical operation, which they call a ‘grammatical virus’.” The insinuation of a virus, in our case a tense/person virus, matches our comparison to the cytokine release syndrome at the end of Sect. 5.2.1 rather well.

30 The explained variation is put in quotation marks since the interpretation of the Nagelkerkes R-square (a so-called pseudo R-squared) is less straightforward than that of comparable methods in linear regression analyses.

31 Importantly, the connection between fusion, which we assume for group 2-speakers and the ‘group’ 3+-speaker, and the realization of multiple copies is also mentioned by Boskovic and Nunes (2007, 50): “Furthermore, given that morphological fusion plays a key role in the licensing of multiple copies, we should in principle expect to find a close-knit relationship between phonetic realization of multiple copies and morphological restrictions.”

32 This confusion is probably caused by the fact that many Brazilians in São Lourenço do Sul and Canguçu, RS de-nasalize the second syllable in *ontem* feeding palatalization of /t/ as [ʧ]. This approximates *ontem* to the palatalized pronunciation of *onde.* (Thanks to Rafael Vetromille-Castro for this hint.)

33 Rizzi (2004, 239) writes: “Evidently, there is a dedicated position for preposed adverbials, which contrary to (certain) Topic positions, is necessarily lower than the position filled by the wh-elements in (50) and (51).”

34 Some Swiss German varieties display, on the surface, exactly the opposite state of affairs as can be seen in (i) and (ii). These tokens from Basel were judged by Isabelle Abt and confirmed for Swiss German from Zurich by Guido Seiler.

**Table:**

(i)	Gescht	hett	(de Maa)	könne	(de Maa)	über	d-Stross	laufe.
	*yesterday*	*had**.3SG.PST.SUBJ*	*the man**.3SG.NOM*	*can**.IPP*	*the man.3SG.NOM*	*over*	*the-street*	*go**.INF*
	‘Yesterday, the man could have crossed the street.’							

(ii)	Gescht	hett	(ich/är)	könne	(*ich/*är)	über	d-Stross	laufe.
	*yesterday*	*had**.1/3SG.PST.SUBJ*	*I/he**.1/3SG.NOM*	*can**.IPP*	*I/he**.1/3SG.NOM*	*over*	*the-street*	*go**.INF*

	‘Yesterday, I/he could have crossed the street.’							For the full-fledged DP *de Maa* ‘the man’, the position in between the TA *hett* and the MV *könne* and the position after the two verbs are both possible. Only the first, but not the second position is available for unstressed subject pronouns regardless of whether they are deictic (1/2SG) or non-deictic (3SG). In Pomerano, exactly the opposite is true. We, therefore, conclude that Swiss German does not allow verb clusters in the CP-domain. *De Maa* after the two verbs in (i) is probably extraposed just like the V3P headed by *laufe* ‘go’. Thus, the MV in Swiss German from Basel/Zurich is probably still in the VP-domain.

35 The parallel to the evolutionary concept of invasibility is obvious. Fitch (2010, 51) defines this concept: “To successfully penetrate a population, a mutant strategy must not only be ‘better’ in some ideal or absolute sense, but must be demonstrably advantageous (receiving a positive differential payoff, on average) relative to the already established strategy or strategies.”
